# The E3 ubiquitin ligase HECTD1 contributes to cell proliferation through an effect on mitosis

**DOI:** 10.1038/s41598-022-16965-y

**Published:** 2022-08-01

**Authors:** Natalie Vaughan, Nico Scholz, Catherine Lindon, Julien D. F. Licchesi

**Affiliations:** 1 Department of Biology and Biochemistry, University of Bath, Claverton Down, Bath, BA2 7AY UK; 2 Department of Pharmacology, University of Cambridge, Tennis Court Road, Cambridge, CB2 1PD UK

**Keywords:** Cell division, Cell signalling, Post-translational modifications

## Abstract

The cell cycle is tightly regulated by protein phosphorylation and ubiquitylation events. During mitosis, the multi-subunit cullin-RING E3 ubiquitin ligase APC/c functions as a molecular switch which signals for one cell to divide into two daughter cells, through the ubiquitylation and proteasomal degradation of mitotic cyclins. The contributions of other E3 ligase families during cell cycle progression remain less well understood. Similarly, the roles of ubiquitin chain types beyond homotypic K48 chains in S-phase or branched K11/K48 chains during mitosis, also remain to be fully determined. Our recent findings that HECTD1 ubiquitin ligase activity assembles branched K29/K48 ubiquitin linkages prompted us to evaluate HECTD1 function during the cell cycle. We used transient knockdown and genetic knockout to show that HECTD1 depletion in HEK293T and HeLa cells decreases cell number and we established that this is mediated through loss of ubiquitin ligase activity. Interestingly, we found that HECTD1 depletion increases the proportion of cells with aligned chromosomes (Prometa/Metaphase) and we confirmed this molecularly using phospho-Histone H3 (Ser28) as a marker of mitosis. Time-lapse microscopy of NEBD to anaphase onset established that HECTD1-depleted cells take on average longer to go through mitosis. In line with this data, HECTD1 depletion reduced the activity of the Spindle Assembly Checkpoint, and BUB3, a component of the Mitosis Checkpoint Complex, was identified as novel HECTD1 interactor. BUB3, BUBR1 or MAD2 protein levels remained unchanged in HECTD1-depleted cells. Overall, this study reveals a novel putative role for HECTD1 during mitosis and warrants further work to elucidate the mechanisms involved.

## Introduction

The Ubiquitin–Proteasome System (UPS) plays important roles during the cell cycle through targeted degradation of key checkpoint proteins. In mitosis, the turnover of cyclins is required in order to commit cells to anaphase and complete cell division. This is achieved through the highly conserved post-translational modifier ubiquitin^1^. Ubiquitin is covalently attached, mostly onto exposed lysines residue of proteins, through a multi-step mechanism which require the activity of an E1-activating enzyme^2–4^, E2-conjugating enzymes and E3 ubiquitin ligases^5,6^. Cullin-RING E3 ubiquitin ligases function as a scaffold, bringing the ubiquitin loaded-E2 into proximity to substrates which enables ubiquitin transfer. In mitosis, the E2-conjugating enzyme UBE2S cooperates with the Anaphase Promoting Complex/cyclosome (APC/c) E3 ligase to extend K11-linked ubiquitin chains onto mitotic substrates, triggering mitotic exit and signalling for the completion of cell division^7–11^.

Ubiquitin can be attached onto protein targets as a single moiety or as a ubiquitin chain. It is assembled into chains through an isopeptide bond between any of its seven lysines (K6, K11, K27, K29, K33, K48 and K63) or the N-terminus (i.e., linear ubiquitin) of an acceptor ubiquitin and the C-terminus of a donor ubiquitin, with all these chain types being found in eukaryotes^12,13^. Therefore, as many as eight linkage types can be used to assemble homotypic, heterotypic, mixed and even branched ubiquitin chains onto protein targets^14^. Ubiquitin polymers assembled through different linkages have different structural and biochemical properties and indeed cellular functions. The cell cycle is perhaps the best example of how these different signals can work in concert for the temporal regulation of molecular mechanisms^15,16^. K48 and K11-linked chains have been shown to function as efficient degradation signals by the UPS, allowing for the timely turnover of cell cycle proteins. For example, K48-linked ubiquitin chains mediate protein degradation of Cyclin Dependent Kinase inhibitor p21, the kinase PLK4 and the phosphatase CDC25 during S-phase. In contrast, the APC/c ubiquitin ligase complex polyubiquitinates cyclin B1, securin as well as aurora kinase A and B with K11-linked chains and thus targets these mitotic proteins for subsequent degradation by the UPS^17–23^.

Although most of our understanding of ubiquitin biology relates to homotypic chains, atypical and branched ubiquitin chains have recently emerged as important signals for cell cycle regulation. For example, branched K11/K48 chains assembled by APC/c have emerged as improved signals for the efficient degradation of mitotic cyclins^8,11,24,25^. In addition to cullin-RING and Skp1-Cul1-F-box protein (SCF) E3s, HECT E3s have also been implicated in cell cycle regulation^26,27^. HECT (Homologous to the E6AP C-terminus) E3 ubiquitin ligases are characterised by intrinsic enzymatic activity contributed by a catalytic cysteine residue located in the C-lobe which accepts ubiquitin from a loaded-E2 through trans-thioesterification prior to transfer onto lysine residue(s) of protein substrates^28–30^. SMURF2 belongs to the NEDD4 subfamily of HECT E3 ubiquitin ligases and was first identified as a regulator of BMP/TGF-β signalling prior to being implicated in chromatin organisation and mitotic regulation. SMURF2 deletion for example impairs the Spindle Assembly Checkpoint (SAC) resulting in defective chromosome alignment/segregation and premature anaphase onset^27^. Further mechanistic studies established SMURF2 uses its ligase activity to assemble non-degradative K63-linked chains onto the Mitosis Checkpoint Complex protein MAD2, thereby protecting it from K48-linked polyubiquitylation and subsequent degradation by the 26S proteasome. SMURF2 has also been implicated at the G2/M transition where its ligase activity stabilises NEDD9 which is required for Aurora A activation and mitotic entry^31^. These studies highlight how the same E3 ubiquitin ligase can act at different stages during the cell cycle and further argues that the ‘ubiquitin code’ in mitosis is more complex than originally thought, with various degradative, non-degradative, typical, and atypical ubiquitin signals involved.

The HECT E3 ubiquitin ligase TRIP12 and its yeast ancestor UFD4 both synthesize homotypic K29-linked ubiquitin onto protein targets and pre-assembled ubiquitin chains^32,33^. HECTD1, the closest homologue to TRIP12, also assembles chains through K29 linkages, although this seems to be in the context of branched K29/K48-linked chains, at least in-vitro^34^. TRIP12, UFD4 and HECTD1 are involved in DNA damage response and chromatin regulation, and HECTD1 has also recently been implicated in histone ubiquitylation during Base Excision Repair (BER)^35–37^. Interestingly, a recent study showed TRIP12 regulates mitotic entry independently of its ubiquitin ligase activity, suggesting that E3 ubiquitin ligase enzymes can have non-enzymatic functions^38^.

Multiple functions have been proposed for HECTD1 during embryonic development for example through the regulation of cell migration and signal transduction pathways such as Wnt, Retinoic acid signalling and NF-κB^39–46^. Different types of ubiquitin chains have been ascribed to HECTD1, with K48-linked ubiquitin chains implicated in the regulation of the focal adhesion protein ACF7 and PIPKIγ90, while K63 has been suggested for its role on HSP90 and the Wnt antagonist Adenomatous polyposis coli (APC)^41,45,47^. Further, HECTD1 has also been implicated in regulating Estrogen Receptor (ER)-mediated transcription. In this mechanism, condensin I and condensin II bind to the ER-α enhancers where they recruit HECTD1, and this leads to the ubiquitin-dependent degradation of the corepressor RIP40 and transcriptional activation of ER-α gene targets^48^. HECTD1 therefore seems to assemble both proteasomal and non-proteasomal ubiquitin signals, and it will be important to further delineate the cellular relevance of its K29/K48 ligase activity.

In this study, we explored HECTD1 function in the context of cell proliferation and found that loss of HECTD1 ubiquitin ligase activity reduces cell proliferation through an effect on mitosis. Our data support recent evidence implicating K29-linked ubiquitylation in mitotic regulation and provide novel insights on the ‘ubiquitin code’^49^.

## Materials and methods

All the methods were performed in accordance with relevant guidelines and regulations.

### Mammalian cell culture

Human embryonic kidney 293 T (HEK293T), HeLa cells, glioblastoma cell lines U87 and U251 were purchased from ATCC. HEK293T HECTD1 knock out and control cells, and HEK293ET were kindly contributed by Dr Mariann Bienz, MRC-LMB Cambridge, UK. HEK and HeLa cells were cultured in Dulbecco’s modified minimum essential medium (DMEM) with GlutaMAX supplement, supplemented with 10% (v/v) Fetal Bovine Serum and 1% (v/v) 10,000 units Penicillin-10 mg/ml Streptomycin, at 37 °C in a humidified incubator with 5% CO_2_^50^. Cells were passaged by incubating with sterile 0.05% EDTA-PBS for 5 min at 37 °C, followed by pelleting the cells at 1000 rpm for 3 min. Cells were then resuspended in supplemented DMEM (with GlutaMAX) and seeded (1/10) in a Nunclon Delta surface-treated (Nunc) 10 cm dish. HECTD1 knock out cells were cultured from passage 4 to passage 35. Both HEK293T HECTD1 KO1 and KO2 have been generated using the same gRNA and these are confirmed individual clones (and not pools)^50^. Glioblastoma cell lines U87 and U251 were cultured in Eagle’s Minimum Essential Media (EMEM) (Sigma-Aldrich, M2279), supplemented with 10% (v/v) Fetal Bovine Serum (Heat-inactivated FBS), 2 mM l-glutamine, MEM 1% (v/v) non-essential amino acids, 1 mM sodium pyruvate, and 1%(v/v) 10,000 units Penicillin-10 mg/ml Streptomycin, at 37 °C in a humidified incubator with 5% CO_2_. U87 and U251 cell lines were passaged as described above. All cell lines were tested for Mycoplasma contamination, using the MycoAlert Mycoplasma Detection Kit (Lonza, Switzerland), as per manufacturer’s instructions.

### Cell synchronisation

To synchronise the cells with RO3306, cells were treated with 9 µM RO3306 for 20 h. To release cells into mitosis, cells synchronised with RO3306 were washed three times in PBS and released into fresh media.

For double thymidine block, cells were treated with 2 mM thymidine (Sigma-Aldrich, T9250) for 18 h, and then washed with PBS once, before adding fresh media containing 25 µM 2′-deoxycytidine (Sigma-Aldrich, D3897) for 9 h. After 9 h, 2 mM thymidine was added for 15 h. Cells were washed three times in PBS and released in fresh media containing 25 µM deoxycytidine for synchronous progression of the cell cycle from the G1/S transition.

For synchronisation using Aphidicolin, cells were cultured in medium with reduced serum (0.5% FBS (v/v)), for 48 h before treatment with 11.8 µM Aphidicolin (Fisher Scientific, 38966-21-1) for 15 h. Cells were washed three times in PBS and released in fresh media for synchronous progression of the cell cycle from the G1/S transition.

#### siRNA transfection

HEK293ET or HeLa were seeded one day prior to transfection into DMEM + 10% (v/v) FBS, without antibiotics present. Twenty-four hours later, HEK293ET/HEK293T cells and HeLa were transfected using Lipofectamine^®^ 2000 or RNAiMAX, respectively (Thermo Fisher Scientific, Waltham, MA, USA). For one well from a 24-well plate 20 pmol of siRNA (HEK293ET) or 25 pmol (HeLa) were transfected in OptiMEM according to the manufacturer’s protocol. For U87 and U251 siRNA transfection, RNAiMAX was used according to the manufacturer’s protocol (Thermo Fisher Scientific).

All siRNAs were obtained from GE Dharmacon including On-TARGETplus Non-Targeting Pool siRNA (D-001810-10-05), On-TARGETplus HECTD1 Individual #06 siRNA (J-007188-06), #07 siRNA (J-007188-07), #08 siRNA (J-007188-08), #09 siRNA (J-007188-09) and On-TARGETplus HECTD1 SMARTpool siRNA (J-007188-00). TRABID siRNA used include ON TARGETplus SIRNA SMARTpool, siRNA siRNA #6 from Dharmacon SMARTpool (GE Healthcare, Dharmacon, Inc., Lafayette, CO, USA) and siRNA #2 from Ref.^51^ (Labelled as TRABID siRNA H2)^34,51^. Oligos were synthesized by Eurofins Scientific (Luxembourg).

### DNA transfection

HEK293ET and HEK293T cells were seeded one day prior to transfection into DMEM + 10% (v/v) FBS, without antibiotics present. Twenty-four hours later, the transfection mix was made by combining branched PEI (MW ~ 25,000) (Sigma-Aldrich, St Louis, MI, USA, 408,727) and DNA at a ratio of 3:1, diluted into 150 mM NaCl. PEI and DNA (250 ng for one well of a 24-well) were individually diluted in 150 mM NaCl, left for 5 min before being combined and then incubated for 15 min at room temperature RT prior to addition to each well.

### Propidium Iodide flow cytometry cell cycle analysis

HeLa cells were seeded at 300,000 cells per well and HEK293ET cells at 500,000 cells per well in 6-well plates, prior to addition of the appropriate cell synchroniser. At each time point, samples were harvested in the following manner. The culture media was removed and saved in an Eppendorf, the cells were then rinsed with PBS, prior to addition of 300 µl of 0.05% Trypsin-EDTA (ThermoFisher Scientific). Once the individual cells were fully detached, the media was added back to generate a single cell suspension. Cells were pelleted at 500 × *g* for 5 min, and the pellet was washed in PBS. Cells were fixed in 69% ethanol, 400 µl ice-cold PBS with 900 µl ice-cold 100% ethanol. Samples were then stored at 4 °C for at least 2 h before staining. Samples were stained and data collected on the same day. Two µg/ml Propidium Iodide (PI) (Thermo Fisher Scientific, P3566) and 100 µg/ml RNase A (Thermo Fisher Scientific, EN0531) were diluted in 500 µl flow cytometry staining buffer (100 mM Tris pH 7.4, 150 mM NaCl, 1 mM CaCl2, 0.5 mM MgCl_2_, 0.1% Nonidet P-40). The samples were then incubated at 37ºC for 30 min before being placed on ice in the dark, just before loading on the BD FACSCanto (BD Biosciences, San Jose, CA, USA). Data were analysed using BD FACSDIVA software V.8.0.1. For cell cycle analysis, histograms were gated at 50 PI-A to calculate the percentage of cells in G1 and 100 PI-A to calculate the % of cells in G2.

For flow cytometry analysis following SAC activation by Nocodazole, HEK293ET cells were first treated with either non-targeting siRNA (NT siRNA) or HECTD1 SMARTpool siRNA (HECTD1 SP) for at least 48 h followed by Nocodazole treatment (18 h, 50 ng/μl). DMSO was used for the control experiment as indicated. Following treatment, cells were harvested and handled as described above.

### Proliferation assay—trypan blue

HEK293ET and HEK293T cells were seeded at 60,000 cells per well in a Poly-l-Lysine coated 24-well plate (Corning Inc., Corning New York, USA, 3524). At each time point cells were trypsinised and resuspended in DMEM + 10% (v/v) FBS, before being mixed in a 1:1 ratio with Trypan Blue solution 0.4% (Thermo Fisher Scientific). Cells were then counted under a light microscope using a haemocytometer. Blue cells indicated dead cells. Counts were carried out in triplicate for each sample over three independent experiments.

### Proliferation assay—CellTiter-Glo assay

HEK293T cells were seeded at 4,000 cells per well into a Poly-l-Lysine coated 96-well clear-bottomed white walled plate (Corning, 3903) in DMEM + 10% (v/v) without antibiotics. 48 h later cells were transfected with either empty vector (EV), full-length mouse HA-Hectd1^WT^, or HA-Hectd1^C2579G^ vector (Gift from Professor Irene Zohn, Children’s National Research Institute, USA), then left for 48 h before measuring the ATP content using CellTiter-Glo assay kit (Promega, Madison, WI, USA, G7570)^47^. The assay was carried out according to the manufacturer’s protocol for a 96-well format and measured using the GloMax Multi Plate Reader (Promega).

### Immunofluorescence

For confocal imaging, cells were plated at an experiment specific density onto Poly-l-Lysine (mol wt 30,000–70,000) (Sigma-Aldrich, 9155) coated Nunc Thermanox 13 mm coverslips (Thermo Fisher Scientific, 1749500) in 12-well plates (Corning, CLS3513). Asynchronous or synchronous cells were then fixed with 4% (w/v) paraformaldehyde and stained. Cells were permeabilized in IF blocking buffer (PBS, 3% BSA, 0.1 Triton X100) for 5 min at RT. Primary and secondary antibodies were incubated in IF blocking buffer (PBS, 3% BSA) for 1 h at RT each. Cells were washed twice with PBS and then counterstained with Hoechst 33342 (Thermo Fisher Scientific, H1399; 1 µg/ml in PBS), followed by two more washes in PBS. Coverslips were mounted with VectaShield Antifade Mounting Medium (Vector Laboratories, Burlingame, CA, USA; H-1000). Samples were then imaged on a LSM Meta 510 confocal microscope (Zeiss, Oberkochen, Germany). Primary antibodies included Anti-α-tubulin (Abcam, Ab7291, 1:1000), Anti-HECTD1 (N-terminus) (Abcam, Ab101992, 1:200), Anti-HA (Roche, Switzerland; HA3F10, 1:1,000). Secondary included Alexa Fluor488 Goat anti-Mouse (Thermo Fisher Scientific’ A11029, 1:500), Alexa Fluor546 Donkey anti-Rabbit (Thermo Fisher Scientific; A10040, 1:500), Alexa Fluor456 Goat anti-Rat (Thermo Fisher Scientific; A11081, 1:500).

### Live cell imaging

For live cell imaging, HEK293ET and HEK293T cells were seeded at 30,000 cells per ml onto Poly-l-Lysine (MW 30,000–70,000) (Sigma-Aldrich, 9155) coated plastic 8-well chamber slides (Ibidi IB-80826). Cells were left overnight in 300 μl/well DMEM + 10% (v/v) FBS, before exchanging the media the following day for Leibovitz’s L-15 media (no phenol red) (Thermo Fisher Scientific, 21083027), with 10% (v/v) FBS. Cells were then filmed over the course of hours at either 2, 3, or 5 min intervals using an Olympus IX81 microscope with a 40× oil immersion objective lens and Hammatsu ORCA-ET Camera for imaging at 37 °C. Micro-Manager was used to acquire and manage the images^52^. Only the DIC channel was used for imaging. A binning of 2 was used for image acquisition, and images were acquired as a stacked TIFF format for downstream analysis. Images were then processed using ImageJ software^53^.

### Western blot analysis

Control asynchronous or synchronised cells were grown in an appropriately sized plate depending on the assay type, then lysed with either RIPA or Triton X-100 lysis buffer supplemented with Pierce Protease Inhibitor Mini Tablets (Thermo Fisher Scientific, 86665) and Pierce Phosphatase Mini Tablets (Thermo Fisher Scientific, 88667). Cell lysates were clarified by centrifugation and samples were denatured at 95 °C for 5 min in NuPage 2X LDS/100 mM DTT sample buffer. Samples were run on NuPAGE Novex 4–12% Bis–Tris Protein Gels, 1.0 mm gels (Thermo Fisher Scientific, NP0322BOX) for 100 min at 140 V in 1X NuPAGE MOPS SDS Running Buffer (Thermo Fisher Scientific, NP0001). Samples were run alongside the PageRuler Prestained Protein Ladder (Thermo Fisher Scientific, 26,616). For immunoblotting, samples were transferred onto Whatman Westran PVDF membrane 0.45 µm using the Bio-Rad Mini Trans-Blot Wet Transfer System (Bio-Rad, Hercules, CA, USA), for 60 min at 100 V in a Wet Transfer system. PVDF membranes were blocked in WB blocking buffer (3% BSA-PBS-Tween 0.1%) for 1 h at RT. Primary antibodies were diluted in WB blocking buffer and incubated either over-night at 4 °C or 1 h at RT. Membranes were then washed in PBS-Tween (0.1%) and then incubated with species-specific horseradish-peroxidase (HRP)-conjugated secondary antibodies diluted in WB blocking buffer for 1 h at RT. Membranes were washed prior to detection with the Pierce ECL Western Blotting Substrate (Thermo Fisher Scientific, 32106). Chemiluminescence was detected using Fusion SL Chemiluminescence and Fluorescence Imager (Vilber Lourmat, France). Quantification of bands was carried out using ImageJ software^53^.

Primary antibodies used include: Anti-HECTD1 (N-terminus) (Abcam, Ab101992, 1/2,500), Anti-Cyclin B1 (Santa Cruz Biotechnology, sc-245, 1/1,000), anti-phospho-Histone H3 (Ser28) (Abcam, Ab10543, 1/1000), anti-β-actin (Sigma-Aldrich, A5441, 1/10,000), anti-CENPF (Abcam, Ab5, 1/1000), anti-BUB3 (Abcam, Ab133699, 1/1000), anti-BUBR1 (Abcam, Ab215351, 1/1000), anti-MAD2 (Bethyl Laboratories, A300-310A, 1/1000), anti-Plk (Santa Cruz Laboratory, F-8/sc-17783, 1/1000), anti-GAPDH (Santa Cruz Laboratory, sc-25779, 1/2000), phospho-Chk2 Thr68 (Cell Signaling Technology, #2661, 1/1000), p21Waf1/Cip1 (Cell Signaling Technology, #2947) and PARP (Cell Signaling Technology, #9542, 1/1000). Secondary antibodies used include: Goat anti-Mouse IgG HRP (Santa Cruz Biotechnology, sc-2054, 1/5000), Goat anti-Rabbit IgG HRP (Santa Cruz Biotechnology, sc-2005, 1/5000), Goat anti-Rat IgG HRP (Santa Cruz Biotechnology, sc-2006, 1/5000), Rabbit anti-Goat IgG HRP (Thermo Fisher, 31402, 1/5000). To enable detection of the same samples with different antibodies, membranes were cut prior to hybridization. Uncropped western blot images are included in the “Supplementary data”, with cropped areas highlighted with a red box.

### GST pulldown

For the enrichment of ubiquitin chains, GST-TRABID NZF 1–3 was used while GST-TRABID NZF 1-3^TY>LV^ (ubiquitin binding deficient mutant) and/or GST were used as control baits. All recombinant proteins were expressed in E. Coli BL21(DE3)RIL and purified a previously described^54^. For each pull down condition, 10 μg of GST tagged protein was bound to Pierce Glutathione Magnetic Agarose Beads (Thermo Fisher, 78,602), for 1 h at RT. Cells in 10 cm^3^ dishes were lysed in 500 μl of GST-TRABID NZF 1–3 IP Lysis Buffer (150 mM NaCl, 50 mM Tris pH 7.4, 5 mM DTT, 2 mM NEM, 10 mM iodoacetamide, 1% v/v Triton X-100) for 20 min before being clarified by centrifugation (13000 rpm for 15 min at 4 °C). 60 μl of lysate was collected for the input sample. For each pull down condition, 110 μl of lysate was added to 50 μl of conjugated beads in 500 μl of Pull-Down Buffer (150 mM NaCl, 50 mM Tris pH 7.4, 5 mM DTT, 2 mM NEM, 10 mM iodoacetamide, 100 μM ZnCl2, 0.1% (v/v) Nonidet P-40, 0.5 mg/ml BSA) overnight at 4 °C . Beads were then washed four times in Wash Buffer (250 mM NaCl, 50 mM Tris pH 7.4, 5 mM DTT, 0.1% (v/v) Nonidet P-40) for 5 min*,* before addition of 2X LDS sample buffer supplemented with 100 mM DTT.

### Mass spectrometry analysis

HEK293T cells were synchronised in late G2 using RO3306 as described above. Four μg of either IgG or HECTD1 antibody (Ab101992) were bound onto Dynabeads^®^ magnetic beads and incubated with either lysates from G2 or M-phase synchronised cells for 1 h at RT. Beads were washed 5 times prior to denaturation in 2xLDS sample buffer/100 mM DTT at 95 °C for 5 min. Samples were then loaded onto a 4–12% Bis–Tris SDS polyacrylamide gel which was then stained with Imperial Protein Stain (ThermoFisher Scientific). Gel lanes were sliced into of 1–2 mm^2^ pieces and following in-gel trypsin digestion followed by liquid chromatography tandem mass spectrometry (LC–MS/MS) using a LTQ Orbitrap Velos (ThermoFisher Scientific) coupled to a nano-ultra performance liquid chromatography (UPLC) system (Dionex, Sunnyvale, CA, USA) as described previously^34^. LC–MS/MS data were searched with the MASCOT program^55^ against the UniPROT protein database. MS/MS data were validated using Scaffold (http://www.proteomesoftware.com). 772 proteins were identified as candidate interactors for endogenous HECTD1. Supplementary Table 1 shows the analysed MS datasets which include 722 proteins with zero unique peptide identified in the IgG control IP (Sample 1) and at least 2 unique peptides in any of the other conditions (i.e. sample 2 or 3).

### Immunoprecipitation assays

For immunoprecipitation studies, endogenous HECTD1 was immunoprecipitated from either asynchronous HEK293ET cells, cells synchronised in G2 with RO3306, cells synchronised in G2 with RO3306 and released in mitosis (10 min and 30 min post-release from RO3306 block), or cells treated with Nocodazole (18 h, 50 ng/ml), as indicated. 4 µg of anti-HECTD1 antibody or normal Rabbit IgG was coupled to Dynabeads magnetic beads protein G (ThermoFisher Scientific, #10003D) for 1 h at RT. Anti-HECTD1-coupled and IgG-coupled beads were washed 3 × times in Triton Lysis Buffer prior to incubation with cell lysates. Cells were lysed on ice for 20 min in Triton Lysis Buffer supplemented with 1X Pierce Protease Inhibitor and 1X Pierce Phosphatase Inhibitor and the lysates was then cleared by centrifugation. HECTD1 or normal Rabbit IgG-coated beads were then incubated for 1 h with cell lysates at RT on a rotating wheel. Magnetic beads from each condition were then washed 5 × times in Triton Lysis Buffer and finally denatured at 95 °C for 5 min in 2X LDS/100 mM DTT. Samples were then resolved on 4–12% SDS PAGE gels and analysed by immunoblotting. Typically, the input ran on a gel represented 1–5% of the total lysate^56^. To enable detection of the same samples with different antibodies, membranes were cut prior to hybridization. Uncropped western blot images are included in the “Supplementary data”, with cropped areas highlighted with a red box.

### Statistical analysis

GraphPad software (GraphPad Prism, La Jolla, CA, USA) was used for data analysis, including mean, standard error values and statistical analysis. Standard error of the mean (S.E.M.) was calculated to quantify the precision of the mean and to compare differences in the means between conditions. S.E.M. was used to consider both the value of the standard deviation and the sample size. Statistical analysis was carried out either using a one-way ANOVA with a Dunnett’s post-test, or a paired student’s t-test as indicated. A one-way ANOVA with a Dunnett’s post-test was used when comparing a series of conditions to a single control condition. A paired student’s t-test was used when comparing two conditions. Relevant p-values are indicated where relevant by *p < 0.05, **p < 0.01 and ***p < 0.001. A p-value of less than 0.05 was considered to be significant.

### High-content microscopy

10,000 cells were seeded into black-walled 96-well plates (Corning, 3340). For HEK293T cells, plates were also coated with 0.1 mg/mL poly-L-lysine (Sigma-Aldrich, P9155) for 30 min. After 24 h, cells were incubated with 10 μM EdU (Invitrogen Click-iT™ EdU Alexa Fluor™ 488 HCS Assay, C10350) for 1 h followed by PFA fixation and click-labelling according to manufacturer’s instructions. Following EdU labelling, cells were immunostained. Cells were blocked with 3% BSA in PBS for 1 h at room temperature. Primary antibodies included phospho-Histone H3 (Ser28) (Abcam, Cambridge, UK; Ab10543, 1/200), phospho-γH2AX S139 (Cell Signaling Technology, Dancers, MA, USA; #9718; 1/200), phospho-Chk2 Thr68 (Cell Signaling Technology, #2661, 1/200). Secondary antibodies anti-Rabbit 555 (Ab150074, 1/500), anti-Rabbit 488 (Ab150073, 1/500) were from Abcam and anti-Rat 555 (A10522, 1/500) was from Invitrogen/Thermo Fisher Scientific. Antibodies were diluted in 3% BSA in PBS and incubated for 1 h at room temperature. Nuclear counterstaining was done using 1X HCS NuclearMask for 30 min (Thermo Fisher Scientific). Plates were imaged using an IN Cell Analyzer 2000 (Cytiva, Marlborough, MA, USA), followed by image processing using CellProfiler v4.2.0 (https://cellprofiler.org) and data analysis using RStudio (R version 4.1.0) and GraphPad Prism v9.1.1. R Core Team (2021). R: A language and environment for statistical computing. R Foundation for Statistical Computing, Vienna, Austria. https://www.R-project.org/. GraphPad Prism version 9.1.1 for Windows, GraphPad Software, San Diego, California USA, www.graphpad.com.

## Results

### HECTD1 ubiquitin ligase activity contributes to cell proliferation

**Figure 1:**
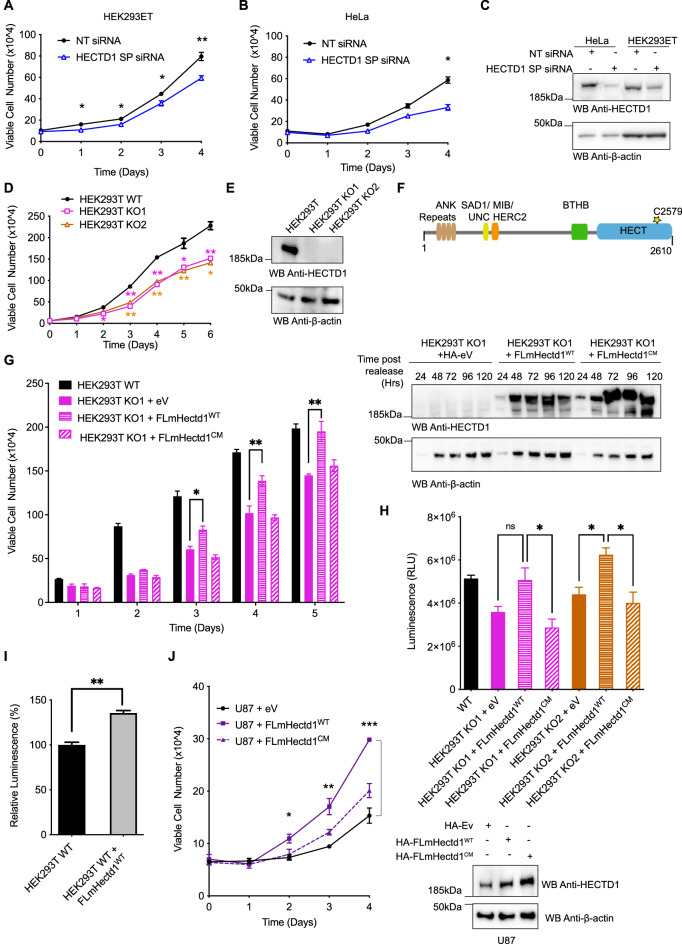
HECTD1 depletion reduces cell proliferation. (**A**, **B**) The Effect of transient siRNA knock down of HECTD1 on cell number was determined using trypan blue exclusion in HEK293ET (**A**) and HeLa (**B**). Data was plotted as mean with error bars that represent ± S.E.M., over three independent experiments (n = 3) defined as three separate transfections, **p < 0.01, *p < 0.05 by paired student’s t-test. (**C**) Immunoblot analysis showing HECTD1 transient knock down using 20 pmol SMARTpool (SP) siRNA in HEK293ET and HeLa following 72 h incubation. HEK293ET and Hela cells were transfected with lipofectamine 2000 or RNAiMAX, respectively. Cells harvested at the indicated timepoint, lysed in RIPA buffer, and lysates were analysed on a 4–12% SDS PAGE followed by western blot analysis using anti-HECTD1, and anti-β-actin antibody as loading control. (**D**) Cell number was also quantified in HEK293T cells and two independent HECTD1 KO cell lines, KO1 and KO2. Data was plotted as mean with error bars that represent ± S.E.M., over three independent experiments (n = 3) defined as three separate transfections, **p < 0.01, *p < 0.05 by paired student’s t-test. (**E**) Immunoblot analysis showing the absence of HECTD1 in HECTD1 KO1 and KO2 cell lysates. (**F**) Domain organisation of mouse Hectd1 highlighting HECTD1 catalytic cysteine C2579. (**G**) Rescue assay showing that re-expression in HEK293T KO1 of HA-FL-mHectd1 wild-type (WT), but not catalytic mutant C2579G or an empty vector control, yields a similar number of cells compared to HEK293T WT cells. Data was plotted as mean with error bars that represent ± S.E.M., over three independent experiments (n = 3) defined as three separate transfections, **p < 0.01, *p < 0.05 by a one-way ANOVA with Dunnett’s post-test. Right panel shows expression levels of HA-tagged constructs. (**H**) A similar trend was also observed using the Cell-Titer-Glo Luminescence cell viability assay. The indicated samples were harvested at 48 h post-transfection prior to analysis. Data plotted as mean with error bars that represent ± S.E.M., over three independent experiments (n = 3) defined as three separate transfections, *p < 0.05 by a one-way ANOVA with Dunnett’s post-test. (**I**) HEK293T WT cells were transfected with HA-FL-mHectd1^WT^ or an HA-empty vector (ev) using PEI. 48 h post-transfection cells were harvested, and cell proliferation measured (relative luminescence) with CellTiter-Glo. Data is plotted as mean with error bars that represent ± S.E.M, over three independent experiments (n = 3), **p < 0.01 by a paired student’s t-test. (**J**) Viable cell count (× 10^4^) for U87 cells transfected with 250 ng either HA-empty vector (Ev), HA-FL-mHectd1^WT^, or HA-FL-mHectd1^C2579G(CM)^ using PEI. Samples were harvested at the indicated times post-transfection and counted using trypan blue to quantify the number of viable cells. Data plotted as mean with error bars that represent ± S.E.M., over three independent experiments (n = 3). ***p < 0.001, **p < 0.01, *p < 0.05 by one-way ANOVA with a Dunnett’s post-test. Right panel shows expression levels of HA-tagged constructs in U87.

**Figure 2:**
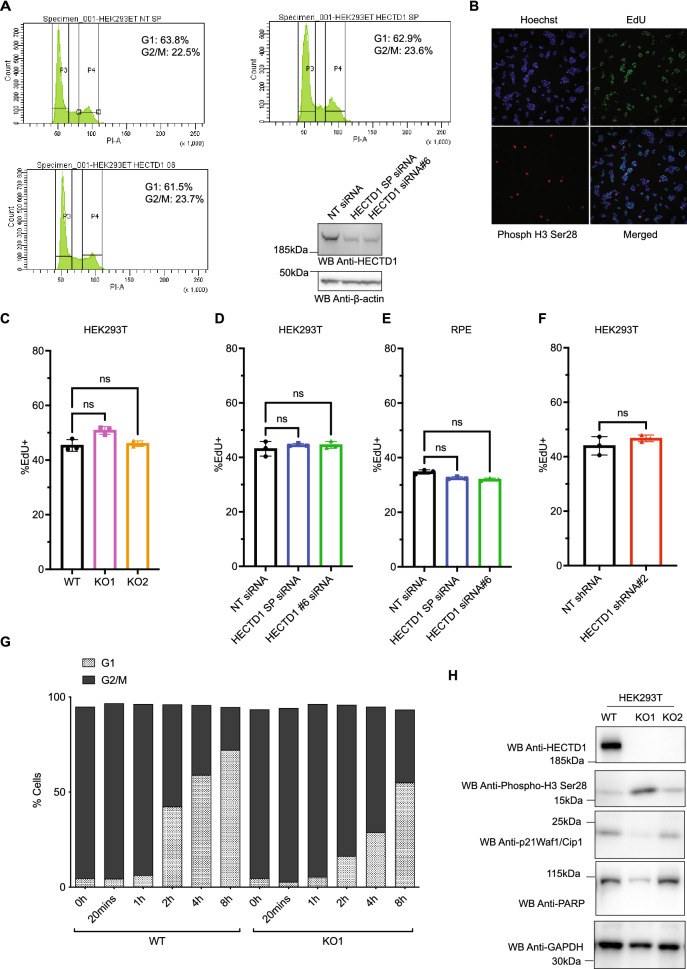
Cell cycle analysis of HECTD1-depleted cells. (**A**) Cell cycle analysis by flow cytometry PI staining in HEK293ET wild-type and HEK293ET cells treated for 48 h with either a non-targeting siRNA (NT siRNA), HECTD1 SMARTpool (SP) siRNA or the individual SMARTpool HECTD1 siRNA #6. (**B**) Representative images of HEK293T cells stained for EdU, phospho-Histone 3 (Ser28), Hoechst and imaged using an IN Cell Analyzer 2000 high-content microscope. Click-EdU staining was used as a readout for cells in S-phase and quantified relative to the total number of cells for: (**C**) HEK293T WT, KO1 and KO2, (**D**) HEK293T siRNA-treated, (**E**) hTERT-RPE siRNA-treated, or (**F**) NT-shRNA or HECTD1-shRNA clone 2. Data plotted as mean with error bars that represent ± S.E.M., over three experiments (n = 3 wells for each condition). Data analysed by unpaired t-test with Kruskal–Wallis. (**G**) Cell cycle analysis by flow cytometry PI staining. HEK293T WT or KO1 cells were synchronised in late G2 with 9 µM RO3306 for 20 h, and then released from block in full media. At each of the indicated timepoints, cells were fixed using 70% ethanol, and stained using 2 µg/ml PI, with 100 µg/ml RNase A, for 30 min at room temperature. Stained samples were then analysed immediately by flow cytometry. Gated population percentages are indicated on each graph. PI-A of 50 is equivalent to 2 N (G1 population), and PI-A of 100 is equivalent to 4 N (G2/M population). Graph showing the percentage of G1 and G2/M populations in HEK293T WT and KO1 cell lines at each time point post RO3306 release. (**H**) Immunoblot analysis of RIPA lysates from HEK293T wild-type, HECTD1 KO1 and KO2 cell lysates. No PARP cleavage nor or a change in p21Waf1/Cip1 levels was observed in HECTD1-depleted cells. In contrast, an increase in the levels of phospho-H3 (Ser28) was detected in both HECTD1 KO lines. GAPDH was used as loading control. To enable detection of the same samples with different antibodies, membranes were cut prior to hybridization. Uncropped western blot images are included in the “Supplementary data”, with cropped areas highlighted with a red box.

While previous studies have established roles for HECTD1 in Wnt signalling, transcription, cell migration and Base Excision Repair (BER), we made the serendipitous discovery that HECTD1-depleted cells grew more slowly than wild-type cells and followed up on this observation. We first used trypan blue dye exclusion to determine the effect of HECTD1 depletion on cell proliferation and found that transient HECTD1 siRNA knockdown (KD) in HEK293ET cells decreased cell number compared to a non-targeting siRNA (Fig. 1A,C). Similar data was obtained in HeLa cells (Fig. 1B,C) and GBM cell lines U87 and U251 (Supp Fig. 1A,B), as well as in two independent HECTD1 KO HEK293T clonal cell lines (Fig. 1D,E). This decrease in cell proliferation was not due to increased cell death as the percentage of viable cells remained similar for up to four days for the siRNA experiment, and 6 days for the KO cells (Supp Fig. 1C–G). To validate this further, we used the CellEvent Caspase 3/7 Green detection reagent which indeed revealed that HECTD1 depletion does not increase caspase mediated cell death under basal conditions (Supp Fig. 1H). We also used PARP cleavage as marker of caspase-mediated cell death but did no detect any PARP cleavage in wild-type nor in HECTD1-depleted HeLa or HEK293T cells (Fig. 2H & data not shown). Therefore, reduced cell proliferation observed upon HECTD1 depletion is not due to increased cell death.

HECTD1 has a conserved C-terminal HECT domain, composed of an N- and C-lobe, with the latter harbouring a conserved cysteine residue which is essential for ubiquitin ligase activity (Fig. 1F): C2579 in human and mouse isoform F8WIE5 (cDNA used in this study) and C2587 in isoform Q69ZR2. To establish whether the observed decrease in cell proliferation was due to loss of HECTD1 ligase activity specifically, we attempted rescue assays using HA-tagged full-length mouse Hectd1 (FL-mHectd1^WT^) or an inactive version of this construct (FL-mHectd1^C2579G^) in HEK293T HECTD1 KO1 cells (Fig. 1G). Re-expression of HA-FL-mHectd1^WT^ but not catalytic-dead HA-FL-mHectd1^C2579G^ recovered cell proliferation to a similar level to wild-type HEK293T cells. These observations were also validated using the CellTiter-Glo luciferase-based proliferation assay which showed a similar trend (Fig. 1H). Ectopic expression of HA-FL-mHectd1^WT^, but not the catalytic mutant, increased cell proliferation in HEK293T and U87 glioblastoma cells (Fig. 1I,J, respectively). Together, these results suggest HECTD1 contributes to cell proliferation through its ubiquitin ligase activity.

### Cell cycle analysis of HECTD1-depleted cells

To establish whether the observed decrease in cell proliferation was due to an effect on the cell cycle, we carried out flow cytometry analysis. Following siRNA-mediated knockdown of HECTD1 in HEK293ET, only a marginal decrease in the proportion of cells in G1 and a similar increase of cells in G2/M was observed (Fig. 2A, Supp Fig. 2A). Since S-phase is not reliably measured by flow cytometry, we used High-Content Microscopy (HCM) to quantify 5-ethynyl-2′-deoxyuridine (EdU) incorporation (Fig. 2B). We observed no change in the proportion of cells in S-phase in HECTD1 KO cells, in HEK293T or hTERT-RPE cells treated with HECTD1 siRNA for 48 h, or in a HEK293T shRNA-HECTD1 clone (Fig. 2C–F). To expand on our observation that depletion of HECTD1 affects cell cycle progression, HEK293T WT and HECTD1 KO1 cells were synchronised in late G2 using 9 µM of RO3306 for 20 h (Supplementary Fig. 2B,C)^57^. The progression of cells following release from G2 block was monitored by flow cytometry at the indicated time points (Fig. 2G, Supplementary Fig. 2D,E). As early as 2 h post RO3306 release, when cells should have already exited M-phase, a higher proportion of cells in G2/M was detected for HECTD1 KO1 cells (79.5%), compared to control wild-type cells (53.7%).

HECTD1 is required for Base Excision Repair (BER) and to show that increase in the G2/M population is not a knock on effect which may have occurred in another cell cycle stage due to DNA damage checkpoints, we probed HECTD1 KO cell lysates for p21Waf1/Cip1 and phospho-Chk2 (Thr68)^36^. BER is mainly carried out during G1-phase, but it has also been reported to take place throughout the cell cycle. Since Phospho-Chk2 (Thr68) and phospho-γH2AX are both upregulated by oxidizing agents, we used H_2_O_2_ or the methylating agent Methyl MethaneSulfonate (MMS) in control experiments (Supplementary Fig. 3A,B)^58,59^. We also probed for p21Waf1/Cip1 or phospho-Chk2 (Thr68) levels by western blot analysis and quantified the number of phospho-Chk2 (Thr68)-positive or phospho-γH2AX-postive cells in HECTD1-depleted cells by HCM but did not detect any change (Fig. 2H, Supplementary Fig. 3C–D). However, we did detect increased phospho-H3 (Ser28) levels in HEK293T HECTD1 KO1 and KO2 (Fig. 2H).

### Phosho-H3 (Ser28) levels are increased in HECTD1-depleted cells

**Figure 3:**
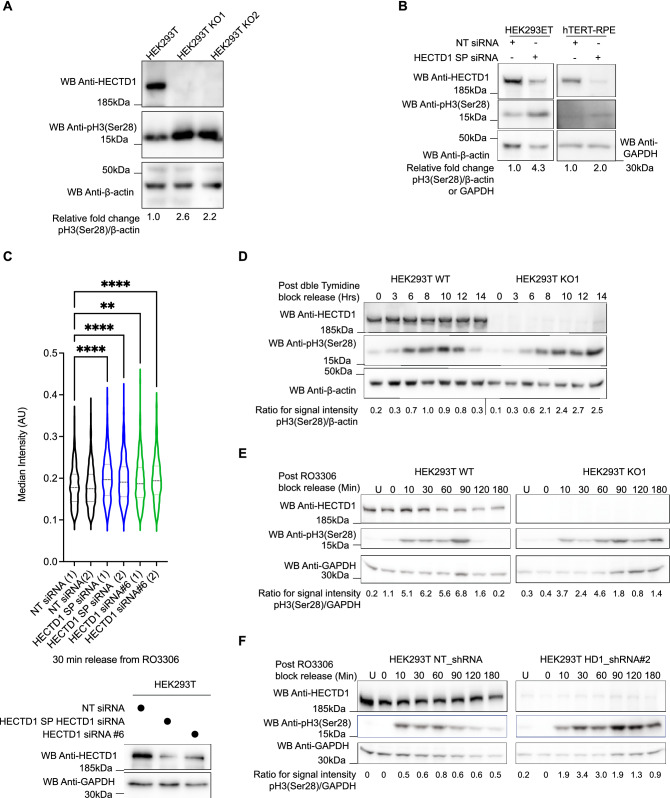
HECTD1 depletion increases phospho-H3 (Ser28) protein levels. (**A**) Phospho-H3 (Ser28) levels were assessed in HEK293T wild-type and KO1 and 2 cell lysates, and changes were quantified and normalized to β-actin. Representative immunoblots of HEK293T KO cells compared to WT control (n = 2). Signal intensity was quantified using ImageJ, ratios for pH3(Ser28)/β-actin were determined and normalised to data from HEK293T cells which was set at 1. (**B**) A similar effect on Phospho-H3 (Ser28) levels was also observed following transient transfection with HECTD1 SMARTpool (SP) siRNA for 72 h in HEK293T (representative data from n = 3) and 48 h in hTERT-RPE cells (n = 1). Signal intensity was quantified using ImageJ, ratios for pH3(Ser28)/β-actin or pH3(Ser28)/GAPDH were determined and normalised to data from NT siRNA-treated HEK293ET or RPE, respectively. (**C**) Quantification of phospho-H3 (Ser28) median signal intensity obtained by high-content microscopy analysis of HEK293T cells treated with siRNA including NT, HECTD1 SMARTpool or HECTD1 siRNA#6. Each condition was set up as two independent wells and data were analysed by One-way Anova. The lower panel shows HECTD1 levels in cells analysed by HCM for this experiment. GAPDH was used as loading control. (**D**) Immunoblots showing a phospho-H3 (Ser28) ‘tail’ in HECTD1-depleted cells compared to control (representative analysis of 2 independent experiments). Cells were treated with 2 mM Thymidine for 18 h, followed by a 9 h release. A second treatment with 2 mM Thymidine for 15 h was carried out, before releasing cells into full media. Samples were harvested at 0, 3, 6, 8, 10, 12, and 14 h post-release. Samples were collected at the indicated timepoints, lysed with RIPA and analysed by immunoblotting using anti-HECTD1, anti-phospho-H3 (Ser28) and anti-β-actin antibodies. Signal intensity was quantified using ImageJ and ratios for pH3(Ser28)/β-actin were determined (**E**, **F**) Immunoblot analysis of cells synchronised using the CDK1 inhibitor RO3306. (**E**) HEK293T WT or KO1 were synchronised in late G2 using 9 µM RO3306 for 20 h, and then released into full media to enter mitosis. Samples were harvested at the indicated timepoints post release, lysed in RIPA, and probed for HECTD1, phospho-H3 (Ser28) and GAPDH (n = 1). (**F**) As in (**E**) but using HEK293T NT-shRNA and HECTD1-shRNA#2 stable cell lines (n = 1). Signal intensity was quantified using ImageJ and ratios for pH3(Ser28)GAPDH were determined. To enable detection of the same samples with different antibodies, membranes were cut prior to hybridization. Uncropped western blot images are included  in the “Supplementary data”, with cropped areas highlighted with a red box.

Phosphorylation of Histone H3 (Ser28) has been used extensively as a reliable marker for early mitosis. Histone H3 is phosphorylated during prophase, and dephosphorylated upon anaphase exit^60^. Given our earlier finding that HECTD1 KO cells showed increased phospho-H3 (Ser28) levels, we quantified and further validated this data using transient siRNA knock down in HEK293T cells (Fig. 3A,B). Phospho-H3 (Ser28) levels were also somewhat increased in hTERT-RPE cells treated with HECTD1 SMARTpool siRNA, in the absence of any detectable change in phospho-Chk2 (Thr68) (Fig. 3B & Supplementary Fig. 3E). Analysis of HCM data confirmed this and revealed that at the population level, signal intensity of phospho-H3 (Ser28)-positive cells is increased in HECTD1 siRNA-treated HEK293T cells compared to non-targeted siRNA treated cells (Supplementary Fig. 4 & Fig. 3C).

To further explore the effect of HECTD1 depletion on mitotic entry and exit, we synchronised HEK293T WT and KO cells at the G1/S checkpoint using double thymidine block and released cells from this block for the indicated time. A signal corresponding to phospho-H3 (Ser28) remained in later time points for HECTD1 KO1 compared to wild-type HEK293T cells (Fig. 3D). Although double thymidine block is one of the most used synchronisers for cell cycle studies, this treatment can also trigger DNA damage which may impact on cell cycle progression. Alternative compounds, including the Cdk4/6 inhibitor Palbociclib, have shown various efficacy at synchronising different cell lines^61^. Therefore, and since our data argue that HECTD1 depletion increases phospho-H3 (Ser28) levels, we monitored progression of cells through mitosis following synchronisation with RO3306^57^. Indeed, we found that phospho-H3 (Ser28) was retained for longer following release from RO3306-induced G2 block in the HECTD1 KO1 cell line as well as the HEK293T HECTD1 shRNA #2 stable cell line compared to controls (Fig. 3E,F, respectively). This data suggests that HECTD1 contributes to cell cycle progression through a yet-to-be identified mechanism in mitosis.

### HECTD1-depleted cells accumulate during mitosis

**Figure 4:**
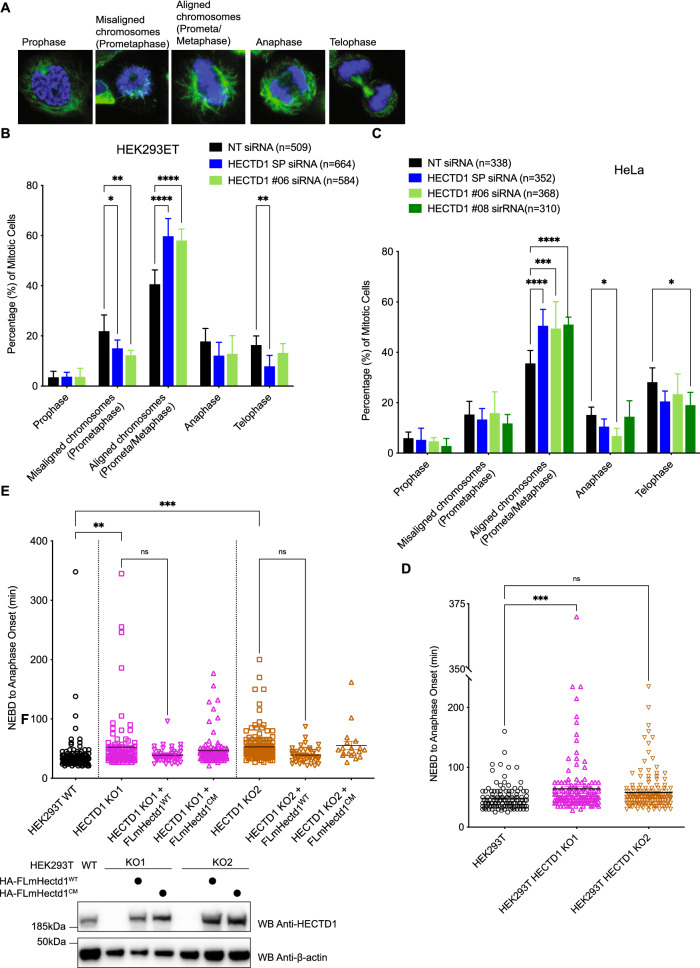
HECTD1 plays a role during mitotic progression. (**A**) Confocal images of HEK293T cells at each mitotic stage which were used for scoring (**B**) and (**C**). Cells were scored according to chromatin morphology based on Hoechst (blue) and α-tubulin (green) staining. Prometaphase refers to cells with misaligned chromosomes while Metaphase (i.e., Prometaphase/Metaphase) refers to cells with aligned chromosomes at the metaphase plate. (**B**) HEK293ET cells and (**C**) HeLa cells were scored according to chromatin morphology or spindle morphology, following 48 h transfection with Non-Targeting (NT) siRNA, HECTD1 SMARTpool (SP) siRNA, SMARTpool individual HECTD1 siRNA #06, or #08. Data plotted as mean with error bars that represent ± S.E.M., over 6 biological repeats (individual transfections). **p < 0.01, ***p < 0.001, and ****p < 0.0001 using a one-way ANOVA with a Dunnett’s post-test. Each HECTD1 siRNA condition was significant when compared to the corresponding NT siRNA control. (**D**) The duration of NEBD to anaphase onset was timed in asynchronous HEK293T WT and KO1 and KO2 cell lines. Vertical scatter plot showing the time taken for individual cells to progress from NEBD to anaphase onset. Error bars represent ± S.E.M., ***p < 0.001, using a one-way ANOVA with a Dunnett’s post-test. Number of cells filmed are as follows, WT = 116, KO1 = 136, and KO2 = 161, filmed over 4 independent experiments. (**E**) Vertical scatter plot showing the time taken (min) for each cell to progress from NEBD to anaphase onset in HEK293T KO1 cells transfected for 48 h with either HA-FL-mHectd1^WT^, or HA-FL-mHectd1^C2579G(CM)^. Lower panel shows expression levels of HA-tagged constructs.

To further explore the impact of HECTD1 depletion on mitosis, we quantified mitotic phenotypes from confocal microscopy images (Fig. 4). Mitotic cells were scored as prophase, misaligned chromosomes (prometaphase), aligned chromosomes (prometaphase/metaphase), anaphase and telophase, based on chromatin and microtubule arrangement (Fig. 4A)^62^. HECTD1 was transiently depleted in HEK293ET and HeLa cells and stained with anti-α-tubulin antibody and Hoechst. In addition to using the HECTD1 SMARTpool we also tested the individual siRNAs #06, #07, #08 and #09 in HEK293ET and HeLa cells (Supplementary Fig. 2A,F, respectively). In HEK293ET cells, 40% of mitotic cells treated with a non-targeting (NT) siRNA showed aligned chromosomes, compared to 57.7% for HECTD1-depleted cells using siRNA #06, and 58.1% for the HECTD1 SMARTpool (SP)-treated cells (Fig. 4B). We observed a similar effect in HeLa cells where 35.7% of cells in the NT siRNA condition were scored as aligned chromosomes, compared to 50.4% for SMARTpool-siRNA, 48.8% for siRNA #06, and 51.7% for siRNA #08 (Fig. 4C). HECTD1-depleted HEK293ET and HeLa cells showed an 18% and 15% increase in the number of cells with aligned chromosomes (i.e., apparent metaphase plate), respectively. Despite the accumulation of mitotic cells following HECTD1 knockdown, we did not observe any obvious or significant effect on mitotic spindles, suggesting that the observed enrichment of HECTD1 depleted cells in metaphase does not translate into mitotic defects in most cells (Supplementary Fig. 5A–C). Although we did detect a slight increase in multinucleated cells and multilobed nuclei in HECTD1-depleted cells, this did not reach statistical significance (Supplementary Fig. 5D–F).

### Loss of HECTD1 ligase activity slows down mitosis

To further establish the role of HECTD1 during mitotic progression, we next used time-lapse microscopy to measure the time taken to progress from Nuclear Envelope Breakdown (NEBD) to anaphase onset. Examination of time-lapse image series of mitotic cells suggested that some cells depleted of HECTD1 showed chromosomes aligned along the equatorial plane for a longer time prior to anaphase onset, compared to wild-type cells (Supplementary Fig. 6A). Time measurement of NEBD to anaphase onset revealed that HEK293T HECTD1 KO1 cells showed a significant mean delay of around 15.5 min and KO2 cells a mean delay of around 9 min compared to the HEK293T control cells (Fig. 4D & Supplementary Fig. 6B). Time-lapse microscopy of HEK293ET cells transiently transfected with HECTD1 SMARTpool or the indicated individual siRNAs also gave a similar trend (Supplementary Fig. 6C,D). At 72 h post-siRNA transfection, HECTD1 SMARTpool showed a mean delay of 21.6 min, and HECTD1 #06 siRNA a significant mean delay of 39.3 min compared to the NT siRNA control. Although siRNA #8 showed no significant mean delay, individual cells still showed a trend towards delayed anaphase onset. Overall, these results suggest that the transient knockdown or genetic knockout of HECTD1 results in a mitotic delay of around 15–30 min on average, representing a 30–60% increase in the duration of mitosis in HEK293T and HEK293ET cells, respectively, which was around 50 min for these cells. Together, these results suggest that some HECTD1-depleted cells progress more slowly from NEBD to anaphase onset which is in line with the observed increase in phospho-H3 (Ser28) levels, and the increase in the proportion of cells with aligned chromosomes.

To ascertain the role of HECTD1 ligase activity in this phenotype, we carried out rescue assays. NEBD to anaphase onset was measured in asynchronous HEK293T KO1 cells transfected with either HA-FL-mouse Hectd1^WT^ or HA-FL-mouse Hectd1^C2579G^ (Fig. 4E & Supplementary Fig. 6E). Re-expression of HA-FL-mHectd1^WT^ but not HA-FL-mHectd1^C2579G^ rescued the mitotic delay phenotype in both HECTD1 KO cell lines (Fig. 4E). HEK293T wild-type cells took on average 37 min to progress from NEBD to anaphase onset. In comparison, both HECTD1 KO1 and KO2 cells showed an increase to 53 min on average. Importantly, HA-FL-mHectd1^WT^ rescued this delay by around 14 min on average. Taken together, our data show HECTD1 ubiquitin ligase activity can rescue the reduced cell proliferation and the delay in NEBD to anaphase onset observed in HECTD1-depleted cells.

### HECTD1 mitotic interactors

**Figure 5:**
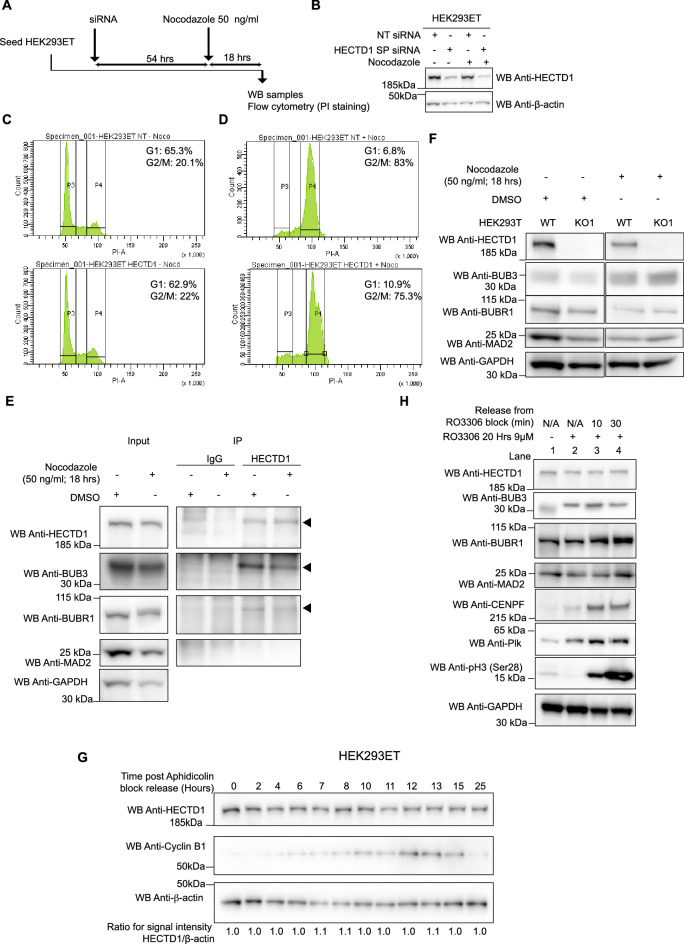
HECTD1 contributes to SAC activation. (**A**) Experimental setup to test for the effect of HECTD1 depletion on SAC activity. (**B**) Immunoblot analysis showing HECTD1 levels following 48 h treatment with HECTD1 SMARTpool (SP) siRNA in HEK293ET cells. β-actin was used as loading control. (**C**, **D**) Cell cycle analysis by flow cytometry PI staining for HEK293ET treated for 48 h with NT (Top) or HECTD1 SP siRNA (Bottom) prior to addition of DMSO (**C**) or Nocodazole (50 ng/ml for 18 h) (**D**), as shown in (**A**). (**E**) Immunoprecipitation assay of endogenous HECTD1 in HEK293T cells showing interaction with endogenous BUB3, but not MAD2 or BUBR1. Normal Rabbit IgG was used for control IP. Representative data of duplicate experiments. Note that the same results were obtained whether cells were asynchronous or arrested in mitosis through nocodazole treatment. (**F**) Immunoblot showing that levels of MCC components BUB3, BUBR1 and MAD2 remains similar in HEK293T WT and HECTD1 KO1 cells. (**G**) Immunoblot showing HECTD1 levels remain similar during S, G2 and M-phase. HEK293ET cells were synchronised in low serum for 48 h before treatment with 4 μg/ml Aphidicolin for 15 h, prior to release in complete media. Cells were harvested at the indicated time points prior to western blot analysis using anti-HECTD1, anti-Cyclin B1 (Sc-245) and anti-β-actin. Signal intensity was quantified using ImageJ and ratios for HECTD1/β-actin were determined. (**H**) HECTD1 levels remain similar during mitosis. HEK293T cells were synchronised in late G2 with RO3306 (Lane 2) or released from RO3306 block into mitosis for 10 min (Lane 3) or 30 min (Lane 4). Phospho-H3 (Ser28) was used to show the effective synchronisation using R03306, while β-actin was used as loading control. Sample from synchronous cells is shown in Lane 1. To enable detection of the same samples with different antibodies, membranes were cut prior to hybridization. Uncropped western blot images are included in the “Supplementary data”, with cropped areas highlighted with a red box.

During mitosis, the Spindle Assembly Checkpoint (SAC) prevents anaphase onset until each kinetochore is attached to the mitotic spindle, ensuring proper chromosome segregation^63^. This mechanism represents one possible explanation for the observed delay in HECTD1-depleted cells. To test this hypothesis, we explored whether SAC activation might be affected in HECTD1-depleted cells. HEK293ET cells treated with either a non-targeting (NT) or with HECTD1 SMARTpool siRNA for 48 h, followed by addition of DMSO or the potent SAC activator, Nocodazole (Fig. 5A,B). Following this treatment, flow cytometry was carried out and cell cycle profiles analysed (Fig. 5C,D). HECTD1 depletion reduced the proportion of cells in G2/M by over 8% upon SAC activation. Therefore, upon SAC activation by nocodazole, HECTD1-depleted cells are less efficient at arresting in mitosis.

The time-resolved interactome of cyclins has revealed important insights with regards to the plasticity of cyclin complexes during cell cycle progression^64^. To try and further explore the mechanisms underlying HECTD1 putative function during mitosis, we carried out a proof-of-principle interactome study to identify HECTD1 candidate interactors (Supplementary Fig. 7A). IgG-coated magnetic Dynabeads® were used to identify non-specific binders using cell lysate from HEK293T cells synchronised in late G2/M using RO3306 (Supplementary Fig. 7B, sample 1). Similarly, HECTD1 antibody-coated Dynabeads® magnetic beads were used with cell lysates obtained from HEK293T cells also synchronised in late G2/M with RO3306 (Sample 2) or from HEK293T cells which had been released from RO3306 block for 20 min and were effectively in M-phase (Sample 3). Pull-down materials from either the IgG control IP or the endogenous HECTD1 IP were then analysed by LC–MS/MS on a LTQ Orbitrap Velos. We identified unique peptides for the Mitotic Checkpoint Complex protein BUB3 in the IPs of endogenous HECTD1 (6 unique peptides for HECTD1 sample 2 and 4 for HECTD1 sample 3) and none in the IgG control IP (Sample 1) (Supplementary Table 1; Supplementary Fig. 7C). BUB3 works as part of a multiprotein complex together with BUBR1, CDC20 and MAD2 to regulate the SAC^65–68^. The Mitotic Checkpoint Complex (MCC) forms at unattached kinetochores, sequestering and inhibiting the APC/c ubiquitin ligase complex and preventing anaphase onset. Since flow cytometry experiments indicated a putative novel role for HECTD1 for full SAC activation, we carried out IP experiments to confirm endogenous HECTD1 indeed interacts with BUB3 but not with MAD2 nor BUBR1 (Fig. 5E). Although a faint signal was observed for BUBR1, this was not reproducible. In contrast, endogenous HECTD1-BUB3 interaction could be detected irrespective of whether cells were asynchronous or synchronised in M-phase using Nocodazole.

The interaction between HECTD1 and BUB3 does not appear to impact on BUB3 levels, which remain similar in HEK293T WT and KO1 cells (Fig. 5F). In support of a novel role for HECTD1 in mitosis, immunofluorescence experiments revealed a pool of HECTD1 is localised to the mitotic spindle. Since the commercially available HECTD1 antibodies which we tested produced either weak or non-specific signals by IF (not shown), we investigated the localization of HA-FL-mHectd1^WT^ in transiently transfected cells (Supplementary Fig. 8A). Confocal microscopy revealed that in interphase cells, including in HEK293T and U87 cells, HA-FL-mHectd1^WT^ is primarily cytosolic (not shown), while it is found at mitotic spindles in cells undergoing mitosis, and this is independent of the ligase activity (Not shown). We also monitored HECTD1 protein levels in synchronised cell populations using Aphidicolin (S-G2-M; Fig. 5G), RO3306 (M-G1; Fig. 5H & Supplementary Fig. 8B) or Nocodazole (M-G1; Supplementary Fig. 8C) which revealed that HECTD1 protein levels are not cell cycle regulated.

### TRABID NZF traps polyubiquitin in each phase of the cell cycle

**Figure 6:**
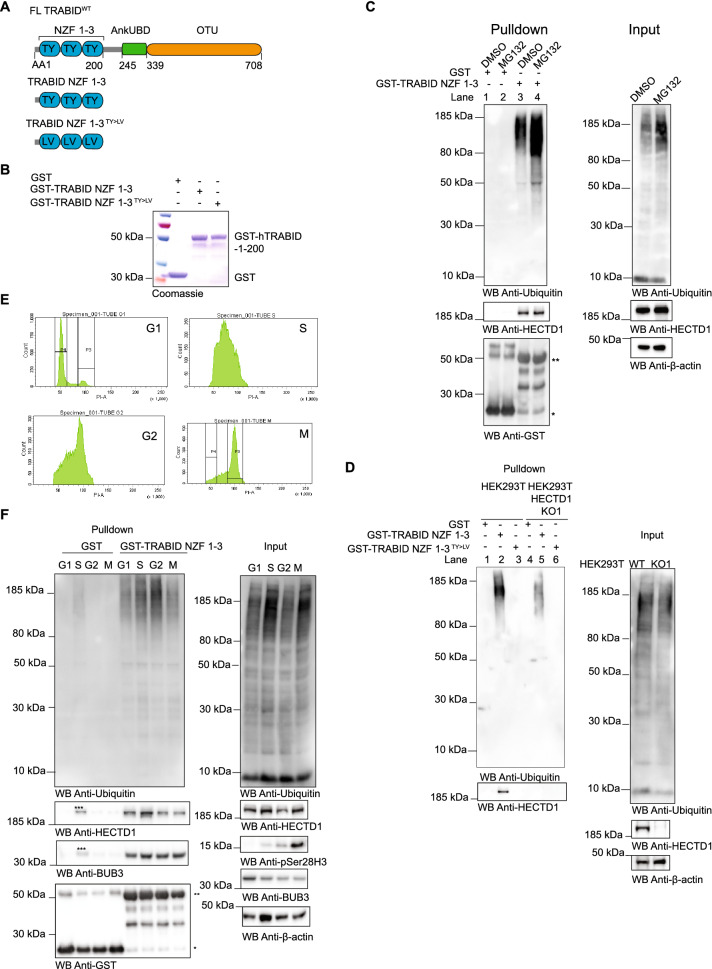
TRABID NZF 1–3 traps ubiquitin chains during cell cycle progression. (**A**) Domain organisation of TRABID showing the AA1-200 region which contains three Npl14 UBDs. GST alone, GST-tagged TRABID NZF 1–3 and the ubiquitin binding deficient TRABID NZF 1–3 TY to LV were produced in E. coli and used as baits for pull-down experiments. (**B**) 5 μg of the indicated recombinant proteins were run on a 4–12% SDS PAGE and stained with Coomassie. (**C**) GST alone or GST-TRABID NZF 1–3 were used as baits in pull-down experiments with cell lysates from asynchronous HEK293T WT cells treated with DMSO or 10 μM MG132 for 6 h prior to cell lysis. Following pull-down with Pierce Glutathione magnetics agarose beads, samples were resolved on 4–12% SDS PAGE, transferred onto PVDF, and probed with anti-Ubiquitin or anti-HECTD1 antibodies. Anti-GST was used as loading control for the baits and β-actin was used as a loading control for lysates. One star (*) indicates GST and (**) corresponds to GST-TRABID NZF 1–3. (**D**) GST-TRABID NZF 1–3 traps ubiquitin and its interaction with HECTD1 requires functional ubiquitin binding domains. Pull-downs and Input were carried out as in (**B**) using lysates from HEK293T WT and HEK293T KO1 mutant cells. (**E**, **F**) Pull-down assay to determine the ability of GST-TRABID NZF 1–3 to pull down endogenous ubiquitin from synchronised HEK293T WT. (**E**) Cell cycle analysis by flow cytometry showing Histograms for each cell cycle phase is shown. PI-A of 50 is equivalent to 2 N (G1 population), and PI-A of 100 is equivalent to 4 N (G2/M population). HEK293T WT cells were synchronised using 4 μg/ml Aphidicolin to enrich for G1 phase cells; 2 mM Thymidine with a 2 h release (double thymidine block) to synchronise cells in S phase; 9 μM RO3306 for 20 h to obtain G2; 9 μM RO3306 for 20 h followed by 20 min release to obtain cells in M phase. (**F**) Immunoblot analysis of pull-downs carried out using lysates from synchronised cell populations and with the indicated GST baits. The membrane for the pull-down experiment was probed for ubiquitin, HECTD1, BUB3, and GST as loading control. Input samples also analysed for the same antibodies as well as Anti-phospho Histone H3 (Ser28) and anti-β-Actin M-phase and loading controls, respectively. * Represents GST, ** GST-TRABID NZF 1–3, and *** reflects uneven loading of the lysate obtained from the S-phase synchronisation. To enable detection of the same samples with different antibodies, membranes were cut prior to hybridization. Uncropped western blot images are included in the “Supplementary data”, with cropped areas highlighted with a red box.

The deubiquitylase TRABID/ZRANB1 preferentially recognizes and processes K29 and K33 linkages over any other linkage types including K63-linked chains^51,54,56^. TRABID three Npl4 zinc finger (NZF) domains act as ubiquitin binding domains, with NZF1 largely responsible for this linkage-specific binding^69–71^. TRABID has emerged as a unique reagent to capture and study atypical ubiquitin chains^51,54,56,69,71^. Our recent work provided compelling evidence that HECTD1 catalytic HECT domain, preferentially assembles branched K29/K48-linked chains^34^. We next used TRABID NZF 1-3 to try and identify K29-linked chains during the cell cycle (Fig. 6A,B). First, we validated the interaction between TRABID and HECTD1 using GST-tagged TRABID NZF 1–3, in line with our previous work (Fig. 6C). Treatment with the proteasomal inhibitor MG132 yielded more ubiquitin species being pulled down by TRABID NZF 1-3. This shows that some of the ubiquitin chains captured by TRABID are involved in proteasomal degradation^72^. We confirmed the signal indicative of interaction between GST-TRABID NZF 1–3 and endogenous HECTD1 was lost in lysates from CRISPR/Cas9 HECTD1 KO cells (Fig. 6D, lane 5 vs. 2). In line with our previous data, the TRABID-HECTD1 interaction was dependent on the ubiquitin binding property of TRABID NZF 1–3, as TY > LV mutations in each of these UBDs abrogated binding to endogenous HECTD1 in pull-down assays (Fig. 6D, lane 3 vs 2)^34,51^. Some polyubiquitylated species could still be detected in the GST-TRABID NZF 1–3 pull down with lysates from HECTD1 KO1 HEK293T cells (Fig. 6D, lane 5 vs. 2). This suggests that some polyubiquitylated species trapped by TRABID NZF 1–3 are likely contributed by other E3s, most likely UBE3C and TRIP12 given these E3s also assemble K29-linked polyubiquitin or perhaps E3s assembling K33-linked chains^30,32,69,71,72^.

Ubiquitin chains have shown some degree of specificity during cell cycle progression, with K48-linked ubiquitin chains being predominant in S-phase while K11-linked ubiquitin chains so far appear to be the main signal for UPS-mediated degradation during mitosis^15^. To further explore whether other atypical ubiquitin chains might be found in specific stages of the cell cycle, we used GST-TRABID NZF 1–3 to capture ubiquitin chains in lysates obtained from HEK293T cells synchronised in G1, S, G2 and M phases (Fig. 6E,F). This revealed that the TRABID-HECTD1 interaction, as well as the polyubiquitylated species recognized by TRABID NZF 1–3, can indeed be captured in all stages of the cell cycle, including during mitosis. To validate the HECTD1-BUB3 interaction using an alternative approach, we also probed for and detected BUB3 in pulldown samples obtained with GST-TRABID NZF 1–3. Taken together, our data reveal a novel interaction between endogenous HECTD1 and BUB3 and suggest that BUB3 might carry ubiquitin chains recognised by TRABID NZF 1–3.

## Discussion

This study was initiated through the fortuitus observation that HECTD1 depletion led to a reduction in cell number, and we determined this was dependent upon the ubiquitin ligase activity of HECTD1. To further elucidate the cellular mechanisms involved, we investigated the impact of loss of HECTD1 on cell cycle progression. Interestingly, a recent study reported that HECTD1 depletion reduces protein synthesis and cell cycle kinetics during Haematopoietic Stem Cells (HSCs) regeneration^73^. Similarly, we found that HECTD1 depletion did not affect cell viability. This study also showed that HECTD1 depletion reduced HSCs in S-phase under stress conditions. However, under basal (i.e., non-stress) conditions, we observed no change in the number of cells in S-phase, which suggests that HECTD1 likely has different effects depending on whether cells are grown under basal or under stress conditions. Flow cytometry revealed only a small increase in the G2/M population upon HECTD1 transient siRNA depletion, but we did observe an increase in the levels of the mitotic marker phospho-H3 (Ser28) in HECTD1-depleted cells.

Our data suggests a new and so far, unreported direct effect of HECTD1 depletion on the cell cycle, specifically during mitosis. In support of this, immunoblotting of synchronised cells, revealed that lysates from HECTD1-depleted cells retained the mitotic marker phospho-H3 (Ser28) for a longer time compared to wild-type cells. HECTD1 contributes to BER and HECTD1 depletion can lead to deficiencies in DNA repair and decreased cell survival in clonogenicity assays^36^. The proposed mechanism appears to be mediated through the ubiquitylation of histones although the type of ubiquitin chains involved remain to be determined. In our assays however, carried out under basal/unchallenged conditions, we did not observe any change in the levels of DNA damage markers p21Waf1/Cip1, phospho-γH2AX or the BER marker phospho-Chk2 (Thr68) between HECTD1-depleted and control cells. This suggested that the effect we observed on mitosis is unlikely due to knock on effects or repair mechanisms taking place at other stages of the cell cycle. To confirm this, we used time-lapse imaging of NEBD to anaphase onset in asynchronous cells as a readout for progression through mitosis. This revealed that HECTD1-depleted cells were indeed slower to progress through mitosis. Importantly, our rescue experiments also indicated that this phenotype could be rescued upon re-expression of full-length wild type but not the catalytic inactive version of mouse Hectd1.

Delay in mitosis, measured by timing NEBD to anaphase onset, can be indicative of the SAC remaining active for a longer time. This leads to APC/c inhibition and delayed anaphase onset. In line with this hypothesis, single cell microscopy analysis revealed an enrichment of cells with aligned chromosomes upon transient siRNA knockdown of HECTD1. We also found that Nocodazole-mediated SAC activation was less efficient in HECTD1-depleted cells compared to control cells, further hinting at a possible role for HECTD1 in the SAC-associated regulation of mitosis. HECTD1 is not the first HECT E3 ubiquitin ligase shown to participate in SAC-activation, with SMURF2 and EDD both previously implicated^27,74^. This raises important questions including how the activity of different E3 ubiquitin ligases is coordinated during SAC activation.

Aurora kinase B and other cell cycle proteins have been identified as candidate HECTD1 interactors in human Embryonic Stem Cells^75^. However, whether and how HECTD1 regulates the Chromosomal Passenger Complex remain to be determined. We identified BUB3 as a putative novel candidate HECTD1 interactor and validated that endogenous HECTD1 does indeed interact with BUB3 but not with BUBR1 or MAD2, two other components of the Mitotic Checkpoint Complex^76^. The HECTD1-BUB3 interaction is likely constitutive since it also takes place in asynchronous cells, and it is independent of SAC activation by Nocodazole. Although HECTD1 depletion appears to reduce the activity of the SAC, protein levels of BUB3, BUBR1 or MAD2 do not appear to be affected. The RepID-CRL4 ubiquitin ligase complex regulates BUB3 levels during mitosis^77^. Interestingly, a recent study proposes that the ubiquitin ligase Ubr5 might be playing a role in the disassembly of the MCC through ubiquitylation of BUB3, BUBR1 and Cdc20 ^78^. It will be important to further evaluate the functional consequences of HECTD1 interaction with BUB3 and delineate how the activity of different E3 ubiquitin ligases might impact on the MCC.

Since our previous data indicated that TRABID depletion might affect HECTD1 protein levels, we tested whether TRABID siRNA would also affect cell cycle progression. We observed a modest increase in the G2/M population by flow cytometry analysis using two different TRABID siRNA (Supplementary Fig. 9A,B). We also measured S-phase progression using EdU incorporation combined with High-Content Microscopy analysis but did not observe any difference between HEK293ET cells treated with NT siRNA or with either of three different TRABID siRNA tested (Supplementary Fig. 9C).

Using TRABID NZF ubiquitin binding domain as bait, we also provide proof-of-principle that ubiquitin chains likely containing K29-linkages can be trapped in different cell cycle stages. Excitingly, a recent study reported the enrichment of K29-linked ubiquitin in stress response and cell cycle regulation using the first K29-specific affimer^49^. In this study, removal of K29-linked ubiquitylation through overexpression of the deubiquitylase TRABID, which preferentially cleaves these linkages, arrested cells in G1. Taken together, this and our own study point at a role for K29 ubiquitylation at various cell cycle stages and warrant further investigation. The K29-specific affimer will be a powerful tool to further explore the role of this linkage type during cell cycle progression, specifically in mitotic cells since K29 is found at the midbody during telophase. In line with our data, HECTD1 and BUB3 were also identified from pulldown and proteomics analysis using the K29-specific affimer. Both TRIP12 and HECTD1 can assemble K29 ubiquitin linkage while TRABID preferentially cleaves this linkage. It will be interesting to further explore whether and how different DUB-E3 pairs regulate K29 ubiquitin signalling during the cell cycle. Mounting evidence suggests K29 linkages have both degradative and nondegradative functions and further work on the composition and topology of these chains is needed.

Interestingly, our data show HECTD1 ubiquitin ligase activity enhances cell proliferation in glioblastoma cells and analysis of two Oncomine datasets suggests *HECTD1* mRNA is found overexpressed in some GBM samples (Supplementary Fig. 10A). The DUB USP15 has been proposed to stabilise HECTD1 levels, which would inhibit Wnt pathway activity and reduce GBM growth^79^. However, in the absence of rescue experiments, it remains unclear whether HECTD1 ligase activity is directly implicated. Furthermore, GBM exhibit high levels of heterogeneity and it will be important to dissect HECTD1 function in GBM subpopulations to provide a deeper mechanistic understanding. In TCGA datasets, high HECTD1 expression is found associated with decreased survival in the mesenchymal GBM subtype and in pancreatic adenocarcinoma (Supplementary Fig. 10B,C)^80^. It will be important to further explore the roles of HECTD1 in these cancers and evaluate its potential as therapeutic target^81^.

## Conclusions

Protein ubiquitylation is a major regulator of cell cycle progression including cell division. The APC/c ubiquitin ligase complex drives the degradation of mitotic cyclins through branched K11/K48-linked ubiquitin chains and this is key to mediate anaphase onset and trigger the completion of mitosis. Yet, whether and how other E3 ubiquitin ligases and ubiquitin signals participate during the cell cycle, including in mitosis, remains less well understood. Our findings show that HECTD1 contributes to cell proliferation through an effect on mitosis. Although the exact mechanisms taking place will need to be further explored, we identified BUB3 as novel HECTD1 interactor. Together with functional assays, our data suggest HECTD1 activity is required for full SAC activation. Our previous work established that the catalytic HECT domain of HECTD1 preferentially assembles branched K29/K48 ubiquitin chains. Since homotypic K63 and K48-linked chains have also been proposed as possible signals assembled by HECTD1 ligase activity, it will be important to establish which is relevant during mitosis. Tandem Ubiquitin Binding Entities and the recently developed ubiquitin K29-specific affimer offer new and exciting opportunities to address these important questions. HECT E3 ligases have been linked with various aspects of tumorigenesis, and future work should also aim to evaluate HECTD1 in cancer, including its potential as therapeutic target.

## Supplementary Information

Supplementary Information 1.

Supplementary Information 2.

## Data Availability

The mass spectrometry proteomics data have been deposited to the ProteomeXchange Consortium via the PRIDE partner repository (https://www.ebi.ac.uk/pride/)^82^. The analysed mass spectrometry proteomics data is included in Supplementary Table 1. Uncropped western blot images are included in the Supplementary data, with cropped areas highlighted with a red box.

## Abbreviations

APC/c: Anaphase Promoting Complex/cyclosome
DUB: Deubiquitylase
HECT: Homologous to the E6AP carboxyl terminus
NEBD: Nuclear envelope breakdown
NZF: Npl4 Zinc Finger
SCF: Skp, Cullin, F-box containing complex
SAC: Spindle assembly checkpoint
UBD: Ubiquitin binding domain
UPS: Ubiquitin-Proteasome System

## References

[1] Glotzer M, Murray AW, Kirschner MW (1991). Cyclin is degraded by the ubiquitin pathway. Nature.

[2] Ciechanover A, Elias S, Heller H, Hershko A (1982). "Covalent affinity" purification of ubiquitin-activating enzyme. J. Biol. Chem..

[3] Haas AL, Warms JV, Hershko A, Rose IA (1982). Ubiquitin-activating enzyme. Mechanism and role in protein-ubiquitin conjugation. J. Biol. Chem..

[4] Mayer A, Gropper R, Schwartz AL, Ciechanover A (1989). Purification, characterization, and rapid inactivation of thermolabile ubiquitin-activating enzyme from the mammalian cell cycle mutant ts85. J. Biol. Chem..

[5] Goebl MG (1988). The yeast cell cycle gene CDC34 encodes a ubiquitin-conjugating enzyme. Science (New York, NY).

[6] Hershko A, Heller H, Elias S, Ciechanover A (1983). Components of ubiquitin-protein ligase system. Resolution, affinity purification, and role in protein breakdown. J. Biol. Chem..

[7] Williamson A (2009). Identification of a physiological E2 module for the human anaphase-promoting complex. Proc. Natl. Acad. Sci. USA.

[8] Matsumoto ML (2010). K11-linked polyubiquitination in cell cycle control revealed by a K11 linkage-specific antibody. Mol. Cell.

[9] Wu T (2010). UBE2S drives elongation of K11-linked ubiquitin chains by the anaphase-promoting complex. Proc. Natl. Acad. Sci. USA.

[10] Wickliffe KE, Lorenz S, Wemmer DE, Kuriyan J, Rape M (2011). The mechanism of linkage-specific ubiquitin chain elongation by a single-subunit E2. Cell.

[11] Min M, Mevissen TE, De Luca M, Komander D, Lindon C (2015). Efficient APC/C substrate degradation in cells undergoing mitotic exit depends on K11 ubiquitin linkages. Mol. Biol. Cell.

[12] Dammer EB (2011). Polyubiquitin linkage profiles in three models of proteolytic stress suggest the etiology of Alzheimer disease. J. Biol. Chem..

[13] Xu P (2009). Quantitative proteomics reveals the function of unconventional ubiquitin chains in proteasomal degradation. Cell.

[14] Kulathu Y, Komander D (2012). Atypical ubiquitylation—The unexplored world of polyubiquitin beyond Lys48 and Lys63 linkages. Nat. Rev. Mol. Cell Biol..

[15] Mocciaro A, Rape M (2012). Emerging regulatory mechanisms in ubiquitin-dependent cell cycle control. J. Cell Sci..

[16] Fournane S, Krupina K, Kleiss C, Sumara I (2012). Decoding ubiquitin for mitosis. Genes Cancer.

[17] Abbas T (2008). PCNA-dependent regulation of p21 ubiquitylation and degradation via the CRL4Cdt2 ubiquitin ligase complex. Genes Dev..

[18] Rogers GC, Rusan NM, Roberts DM, Peifer M, Rogers SL (2009). The SCF Slimb ubiquitin ligase regulates Plk4/Sak levels to block centriole reduplication. J. Cell Biol..

[19] Jin J (2003). SCFbeta-TRCP links Chk1 signaling to degradation of the Cdc25A protein phosphatase. Genes Dev..

[20] Clute P, Pines J (1999). Temporal and spatial control of cyclin B1 destruction in metaphase. Nat. Cell Biol..

[21] Jin L, Williamson A, Banerjee S, Philipp I, Rape M (2008). Mechanism of ubiquitin-chain formation by the human anaphase-promoting complex. Cell.

[22] Hagting A (2002). Human securin proteolysis is controlled by the spindle checkpoint and reveals when the APC/C switches from activation by Cdc20 to Cdh1. J. Cell Biol..

[23] Floyd S, Pines J, Lindon C (2008). APC/C Cdh1 targets aurora kinase to control reorganization of the mitotic spindle at anaphase. Curr. Biol..

[24] Meyer H-J, Rape M (2014). Enhanced protein degradation by branched ubiquitin chains. Cell.

[25] Yau RG (2017). Assembly and function of heterotypic ubiquitin chains in cell-cycle and protein quality control. Cell.

[26] D'Angiolella V (2010). SCF(Cyclin F) controls centrosome homeostasis and mitotic fidelity through CP110 degradation. Nature.

[27] Osmundson EC (2008). The HECT E3 ligase Smurf2 is required for Mad2-dependent spindle assembly checkpoint. J. Cell Biol..

[28] Kim HC, Huibregtse JM (2009). Polyubiquitination by HECT E3s and the determinants of chain type specificity. Mol. Cell. Biol..

[29] You J, Pickart CM (2001). A HECT domain E3 enzyme assembles novel polyubiquitin chains. J. Biol. Chem..

[30] Wang M, Cheng D, Peng J, Pickart CM (2006). Molecular determinants of polyubiquitin linkage selection by an HECT ubiquitin ligase. EMBO J..

[31] Moore FE (2010). The WW-HECT protein Smurf2 interacts with the Docking Protein NEDD9/HEF1 for Aurora A activation. Cell Div..

[32] Kaiho-Soma A (2021). TRIP12 promotes small-molecule-induced degradation through K29/K48-branched ubiquitin chains. Mol. Cell.

[33] Liu C, Liu W, Ye Y, Li W (2017). Ufd2p synthesizes branched ubiquitin chains to promote the degradation of substrates modified with atypical chains. Nat. Commun..

[34] Harris LD (2021). The deubiquitinase TRABID stabilizes the K29/K48-specific E3 ubiquitin ligase HECTD1. J. Biol. Chem..

[35] Ackermann L (2016). E4 ligase-specific ubiquitination hubs coordinate DNA double-strand-break repair and apoptosis. Nat. Struct. Mol. Biol..

[36] Bennett L, Madders E, Parsons JL (2020). HECTD1 promotes base excision repair in nucleosomes through chromatin remodelling. Nucleic Acids Res..

[37] Gudjonsson T (2012). TRIP12 and UBR5 suppress spreading of chromatin ubiquitylation at damaged chromosomes. Cell.

[38] Larrieu D (2020). The E3 ubiquitin ligase TRIP12 participates in cell cycle progression and chromosome stability. Sci. Rep..

[39] Zohn IE, Anderson KV, Niswander L (2007). The Hectd1 ubiquitin ligase is required for development of the head mesenchyme and neural tube closure. Dev. Biol..

[40] D'Alonzo D (2019). Hectd1 is essential for embryogenesis in mice. Gene Expr. Patterns.

[41] Tran H (2013). HectD1 E3 ligase modifies adenomatous polyposis coli (APC) with polyubiquitin to promote the APC-axin interaction. J. Biol. Chem..

[42] Niehrs C, Acebron SP (2012). Mitotic and mitogenic Wnt signalling. EMBO J..

[43] Schlesinger A, Shelton CA, Maloof JN, Meneghini M, Bowerman B (1999). Wnt pathway components orient a mitotic spindle in the early *Caenorhabditis elegans* embryo without requiring gene transcription in the responding cell. Genes Dev..

[44] Shen X (2017). HECTD1 controls the protein level of IQGAP1 to regulate the dynamics of adhesive structures. Cell Commun. Signal.

[45] Li X (2013). Ubiquitylation of phosphatidylinositol 4-phosphate 5-kinase type I γ by HECTD1 regulates focal adhesion dynamics and cell migration. J. Cell Sci..

[46] Duhamel S (2018). The E3 ubiquitin ligase HectD1 suppresses EMT and metastasis by targeting the +TIP ACF7 for degradation. Cell Rep..

[47] Sarkar AA, Zohn IE (2012). Hectd1 regulates intracellular localization and secretion of Hsp90 to control cellular behavior of the cranial mesenchyme. J. Cell Biol..

[48] Li W (2015). Condensin I and II complexes license full estrogen receptor α-dependent enhancer activation. Mol. Cell.

[49] Yu Y (2021). K29-linked ubiquitin signaling regulates proteotoxic stress response and cell cycle. Nat. Chem. Biol..

[50] Flack JE, Mieszczanek J, Novcic N, Bienz M (2017). Wnt-dependent inactivation of the Groucho/TLE Co-repressor by the HECT E3 Ubiquitin Ligase Hyd/UBR5. Mol. Cell.

[51] Tran H, Hamada F, Schwarz-Romond T, Bienz M (2008). Trabid, a new positive regulator of Wnt-induced transcription with preference for binding and cleaving K63-linked ubiquitin chains. Genes Dev..

[52] Edelstein AD (2014). Advanced methods of microscope control using muManager software. J. Biol. Methods.

[53] Schneider CA, Rasband WS, Eliceiri KW (2012). NIH Image to ImageJ: 25 years of image analysis. Nat. Methods.

[54] Komander D (2009). Molecular discrimination of structurally equivalent Lys 63-linked and linear polyubiquitin chains. EMBO Rep..

[55] Perkins DN, Pappin DJ, Creasy DM, Cottrell JS (1999). Probability-based protein identification by searching sequence databases using mass spectrometry data. Electrophoresis.

[56] Licchesi JD (2011). An ankyrin-repeat ubiquitin-binding domain determines TRABID's specificity for atypical ubiquitin chains. Nat. Struct. Mol. Biol..

[57] Vassilev LT (2006). Selective small-molecule inhibitor reveals critical mitotic functions of human CDK1. Proc. Natl. Acad. Sci. USA.

[58] Chen Q (2021). ADP-ribosylation of histone variant H2AX promotes base excision repair. EMBO J..

[59] Chou WC (2008). Chk2-dependent phosphorylation of XRCC1 in the DNA damage response promotes base excision repair. EMBO J..

[60] Gurley LR, D'Anna JA, Barham SS, Deaven LL, Tobey RA (1978). Histone phosphorylation and chromatin structure during mitosis in Chinese hamster cells. Eur. J. Biochem..

[61] Trotter EW, Hagan IM (2020). Release from cell cycle arrest with Cdk4/6 inhibitors generates highly synchronized cell cycle progression in human cell culture. Open Biol..

[62] Pines J, Rieder CL (2001). Re-staging mitosis: a contemporary view of mitotic progression. Nat. Cell Biol..

[63] Murray A (1994). Cell cycle checkpoints. Curr. Opin. Cell Biol..

[64] Pagliuca FW (2011). Quantitative proteomics reveals the basis for the biochemical specificity of the cell-cycle machinery. Mol. Cell.

[65] Fang G, Yu H, Kirschner MW (1998). The checkpoint protein MAD2 and the mitotic regulator CDC20 form a ternary complex with the anaphase-promoting complex to control anaphase initiation. Genes Dev..

[66] Sudakin V, Chan GK, Yen TJ (2001). Checkpoint inhibition of the APC/C in HeLa cells is mediated by a complex of BUBR1, BUB3, CDC20, and MAD2. J. Cell Biol..

[67] Brady DM, Hardwick KG (2000). Complex formation between Mad1p, Bub1p and Bub3p is crucial for spindle checkpoint function. Curr. Biol..

[68] Hardwick KG, Johnston RC, Smith DL, Murray AW (2000). MAD3 encodes a novel component of the spindle checkpoint which interacts with Bub3p, Cdc20p, and Mad2p. J. Cell Biol..

[69] Kristariyanto YA (2015). K29-selective ubiquitin binding domain reveals structural basis of specificity and heterotypic nature of k29 polyubiquitin. Mol. Cell.

[70] Kristariyanto YA (2015). Assembly and structure of Lys33-linked polyubiquitin reveals distinct conformations. Biochem. J..

[71] Michel MA (2015). Assembly and specific recognition of k29- and k33-linked polyubiquitin. Mol. Cell.

[72] Crowe SO, Rana A, Deol KK, Ge Y, Strieter ER (2017). Ubiquitin chain enrichment middle-down mass spectrometry enables characterization of branched ubiquitin chains in cellulo. Anal. Chem..

[73] Lv K (2021). HectD1 controls hematopoietic stem cell regeneration by coordinating ribosome assembly and protein synthesis. Cell Stem Cell.

[74] Scialpi F, Mellis D, Ditzel M (2015). EDD, a ubiquitin-protein ligase of the N-end rule pathway, associates with spindle assembly checkpoint components and regulates the mitotic response to nocodazole. J. Biol. Chem..

[75] Saez I, Koyuncu S, Gutierrez-Garcia R, Dieterich C, Vilchez D (2018). Insights into the ubiquitin-proteasome system of human embryonic stem cells. Sci. Rep..

[76] Musacchio A (2015). The molecular biology of spindle assembly checkpoint signaling dynamics. Curr. Biol..

[77] Jang SM (2020). The RepID-CRL4 ubiquitin ligase complex regulates metaphase to anaphase transition via BUB3 degradation. Nat. Commun..

[78] Kaisari S (2022). Role of ubiquitin-protein ligase UBR5 in the disassembly of mitotic checkpoint complexes. Proc. Natl. Acad. Sci. USA.

[79] Oikonomaki M, Bady P, Hegi ME (2017). Ubiquitin Specific Peptidase 15 (USP15) suppresses glioblastoma cell growth via stabilization of HECTD1 E3 ligase attenuating WNT pathway activity. Oncotarget.

[80] Anaya J, Reon B, Chen WM, Bekiranov S, Dutta A (2015). A pan-cancer analysis of prognostic genes. PeerJ.

[81] Scholz N, Kurian KM, Siebzehnrubl FA, Licchesi JDF (2020). Targeting the ubiquitin system in glioblastoma. Front. Oncol..

[82] Perez-Riverol Y (2019). The PRIDE database and related tools and resources in 2019: Improving support for quantification data. Nucleic Acids Res..

