# Supplementary material for: The E3 ubiquitin ligase HECTD1 contributes to cell proliferation through an effect on mitosis
Source: Sci Rep. 2022 Aug 1;12:13160. doi: 10.1038/s41598-022-16965-y (PMC9343455; doi:10.1038/s41598-022-16965-y)
Supplement: Supplementary file 2 — Supplementary Information 2. [file 41598_2022_16965_MOESM2_ESM.docx]

**The E3 ubiquitin ligase HECTD1 contributes to cell proliferation through an effect on mitosis**

Natalie Vaughan^1#^, Nico Scholz^1#^, Catherine Lindon^2^ and Julien D. F. Licchesi^1, *^

^1^ Department of Biology & Biochemistry, University of Bath, Claverton Down, Bath, BA2 7AY, United Kingdom

^2^ Department of Pharmacology, University of Cambridge, Tennis Court Road, Cambridge CB2 1PD, United Kingdom

# These authors contributed equally

* Correspondence to: Julien DF Licchesi, Department of Biology & Biochemistry, University of Bath, Claverton Down, Bath, BA2 7AY, UK. +44(0)1225 386 287; j.licchesi@bath.ac.uk

**Supplementary Figures**

**
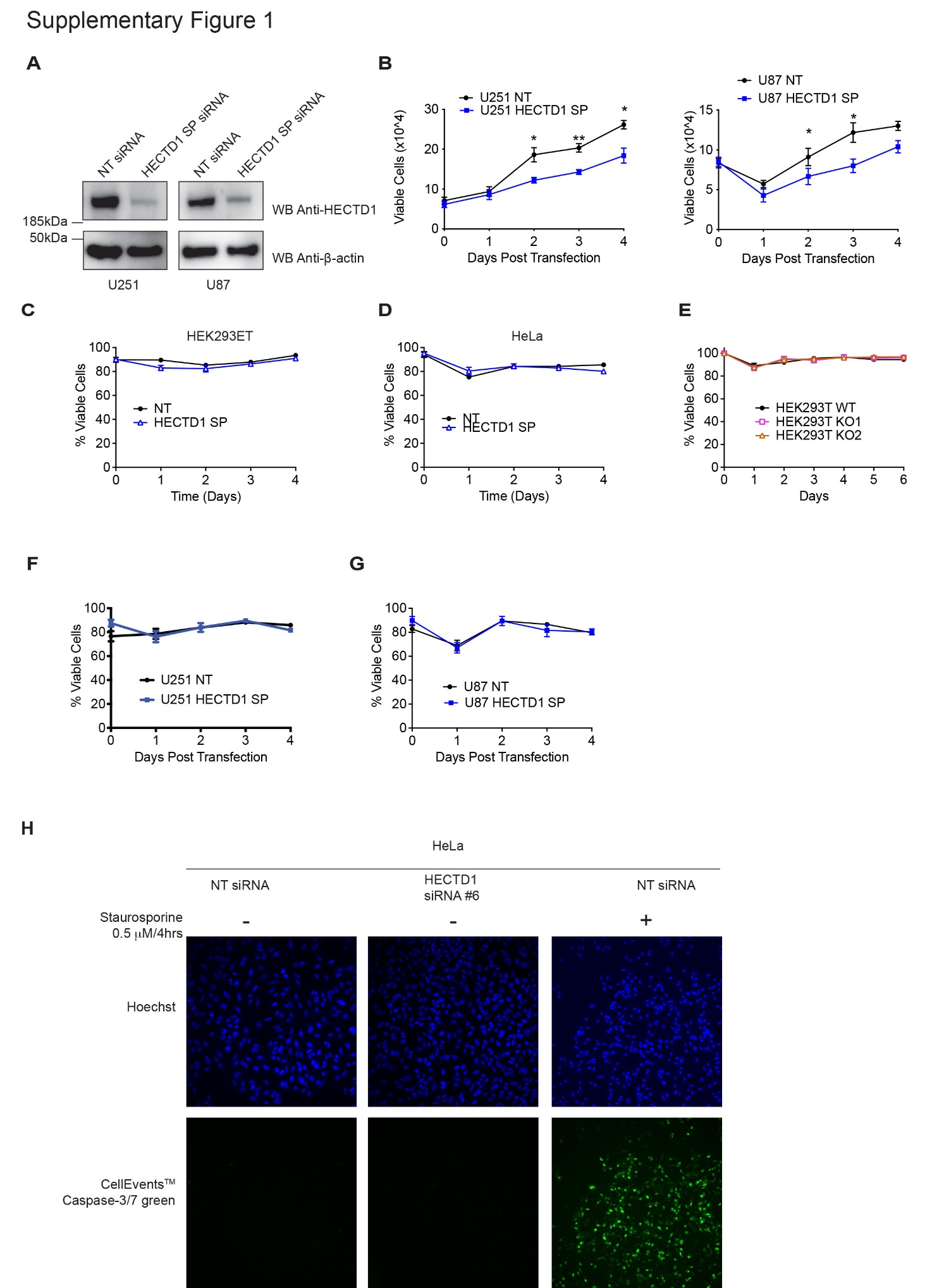
**

**Supplementary Figure 1. HECTD1 depletion does not reduce cell viability**

**A)** Immunoblot showing HECTD1 knock down efficiency in U251 and U87 cells 48 hrs post siRNA treatment. **B)** Viable cell count (x10^4^) is shown for U87 and U251 treated with either Non-Targeting (NT) or HECTD1 SMARTpool (SP) siRNA. Data plotted as mean with error bars that represent ±S.E.M., over three independent experiments (n=3), **p<0.01, *p<0.05 by paired student’s t-test. **C-G**) Trypan blue exclusion test was used to determine cell viability. Data plotted as mean of viable cells (%) with error bars that represent ±S.E.M., over three independent experiments (n=3), **p<0.01, *p<0.05 by paired student’s t-test. (**C**) HEK293ET, **(D)** HeLa, (**E**) HECTD1 KO1 and KO2, (**F**) U251 and (**G**) U87. **H**) Microscopy images obtained using an EVOS Cell Imaging System showing CellEvent Caspase-3/7 green detection reagent as a marker of apoptotic cell death. HeLa cells were incubated with NT or HECTD1 SP siRNA for 48 hrs, prior to detection with CellEvent Caspase-3/7 green reagent. As a positive control for this assay, HeLa cells were treated with 0.5 μM of Staurosporine for 4 hrs to trigger caspase-mediated apoptotic cell death as shown by the increased number of Green-positive cells.

**
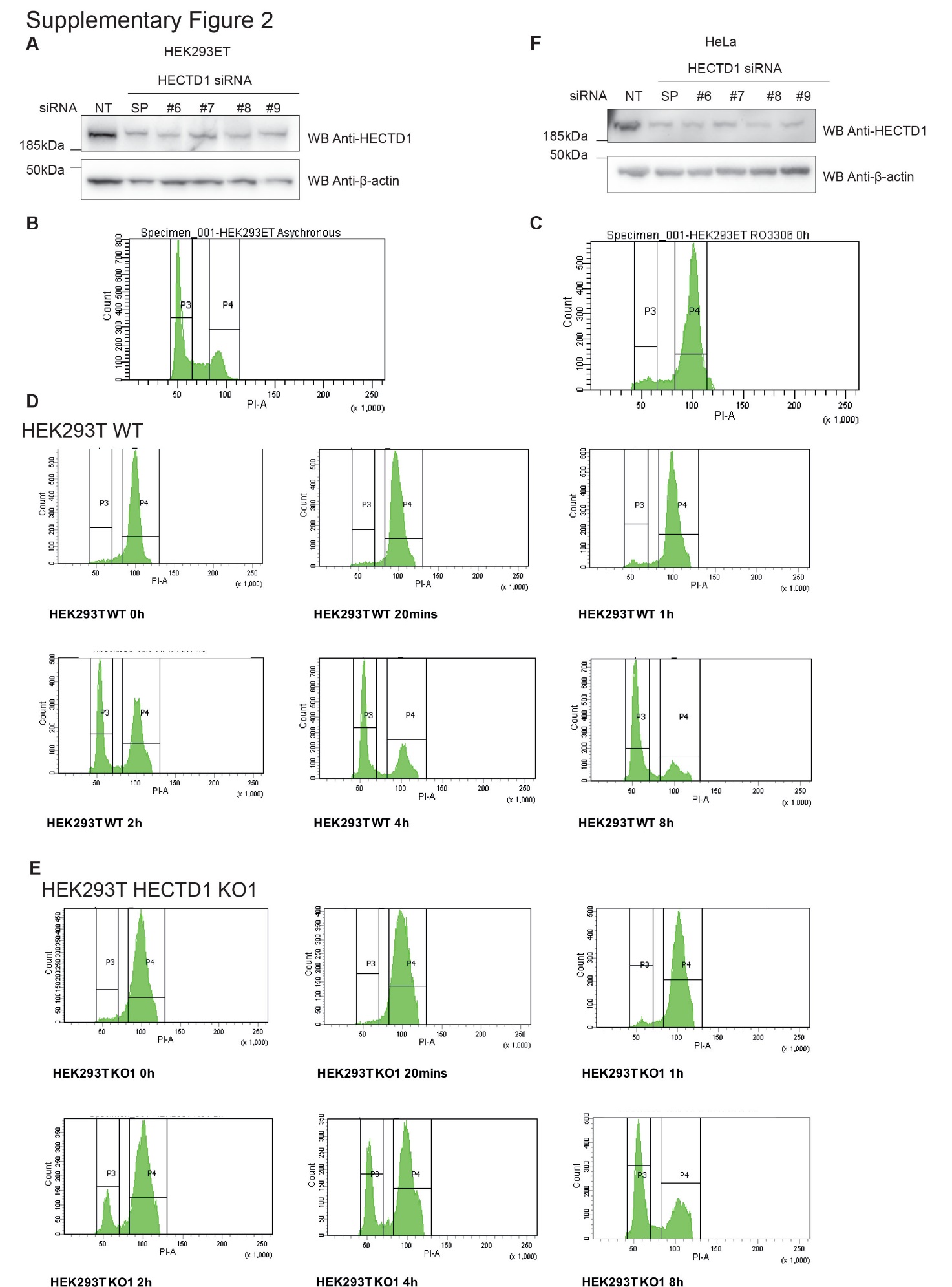
**

**Supplementary Figure 2. Flow cytometry cell cycle analysis of wild-type and HECTD1-depleted cells synchronised in late G2 using RO3306**

**A**) Immunoblotting showing the efficacy of HECTD1 siRNA-mediated knockdown in HEK293ET cells. Cells were transiently transfected for 48 hrs using 10 pmol, 20 pmol, or 40 pmol HECTD1 (SMARTpool, SP) siRNA with Lipofectamine 2000 per well of a 24-well plate. **B & C**) Flow cytometry cell cycle analysis of asynchronous HEK293T cells (**B**), or cells synchronised in late G2 with 9 µM RO3306 for 20 hrs. (**C**). Cells were fixed using 70% ethanol, and stained using 2 µg/ml PI, with 100 µg/ml RNase A, for 30 min at room temperature. Stained samples were then analysed immediately by flow cytometry. Gated population percentages are indicated on each graph. PI-A of 50 is equivalent to 2N (G1 population), and PI-A of 100 is equivalent to 4N (G2/M population). PI-A of 50 is equivalent to 2N (G1 population), and PI-A of 100 is equivalent to 4N (G2/M population). **D, E**) Flow cytometry cell cycle analysis of HEK293T WT (**D**) and HECTD1 KO1 (**E**) following release from RO3306 block. Cells were released in full media prior to processing at the indicated time points (20 min, 1 h, 2 hr, 4 h, 8 h). **F**) Immunoblotting showing the efficacy of HECTD1 siRNA-mediated knockdown in HeLa cells using RNAiMAX as transfection reagent.


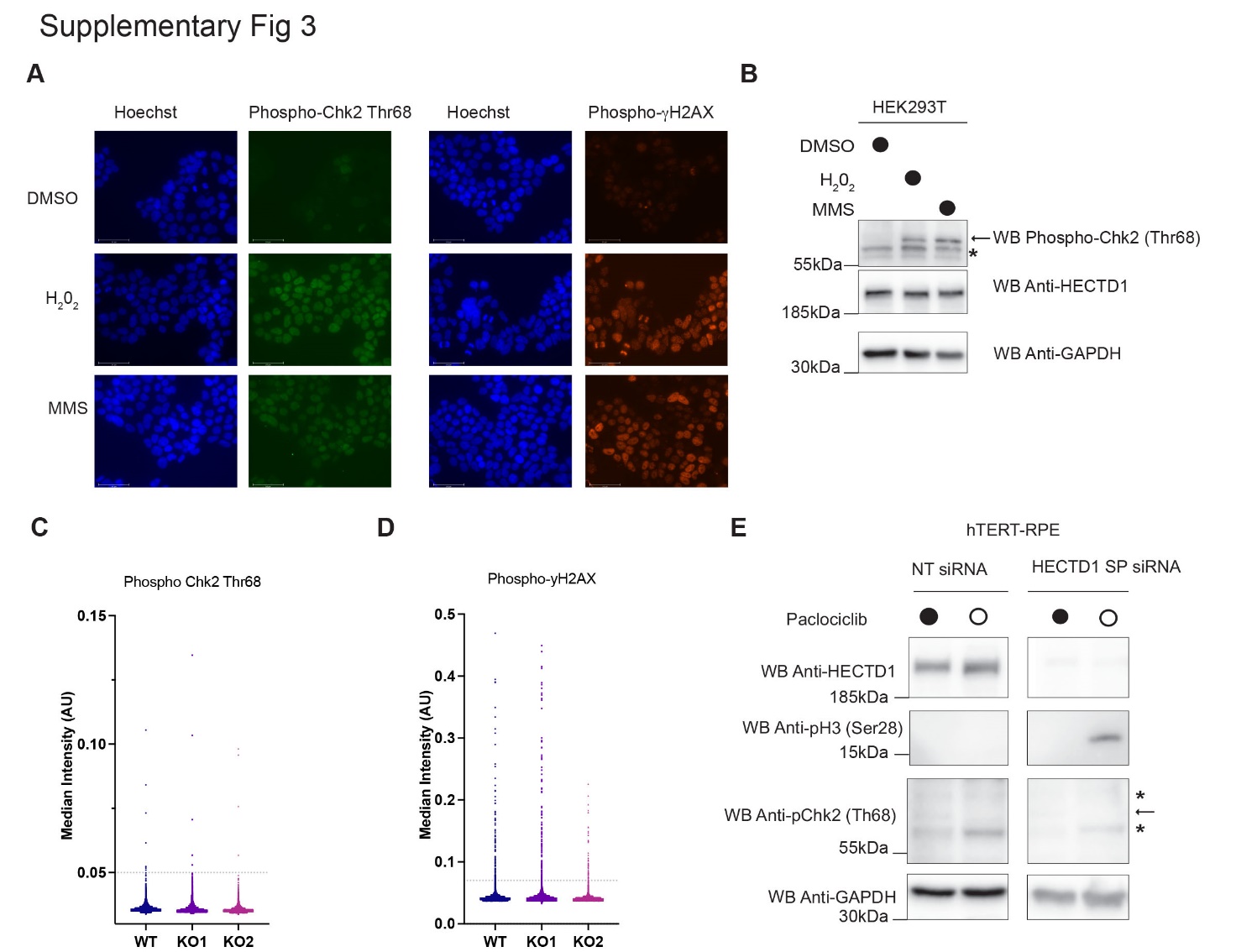


**Supplementary Figure 3. HECTD1 depletion does not increase expression of DNA damage markers**

**A)** Microscopy images obtained using an EVOS cell imaging system validating the detection DNA damage markers phospho-Chk2 (Thr68) and phospho-γH2AX. HEK293T cells were treated with 0.1 mM H_2_0_2_ or 0.3 mg/ml of Methyl MethaneSulfonate (MMS) for 30 min prior to fixation with 4% PFA and immunostaining using phospho-Chk2 (Thr68) and phospho-γH2AX antibodies^1^. Alexa Fluor546 Donkey anti-Rabbit was used as secondary antibody and Hoechst as nuclear stain and then mounted with VectaShield Antifade Mounting Medium. **B**) HEK293T cells were treated as in A) but lysed using RIPA buffer and analysed by immunoblotting using phospho-Chk2 (Thr68). **C-D**) High-content microscopy analysis of (**C**) phospho-Chk2 (Thr68) and (**D**) phospho-γH2AX-positive cells in HEK293T wild-type, HECTD1 KO1 and KO2 cell lines. No significant difference was observed. **E**) Immunoblotting showing the increase in phospho-H3 (Ser28) levels upon siRNA mediated knockdown of HECTD1 in hTERT-RPE cells. This increase did not coincide with an increase in phospho-Chk2 (Thr68). Palbociclib (150 nM for 12 hrs) was used as control since it synchronises cells in G1^2^. Arrow indicates phospho-Chk2 (Thr68)-specific signal and a star (*) non-specific signals.


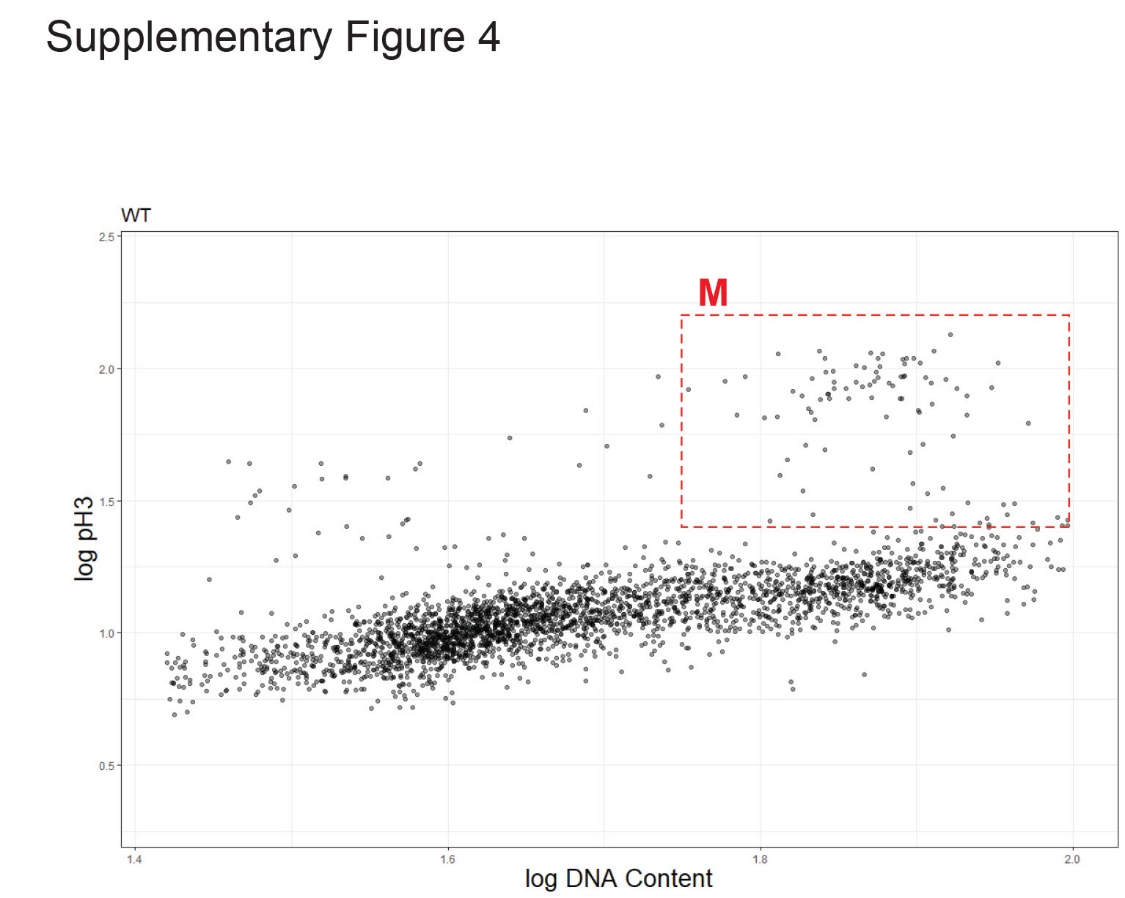


**Supplementary Figure 4. Quantification of phospho-H3 (Ser28)-positive cells**

**A)** Representative analysis of high content microscopy data obtained using anti-phospho-H3 (Ser28) antibody and Hoechst signal intensity to identify cells in M-phase.


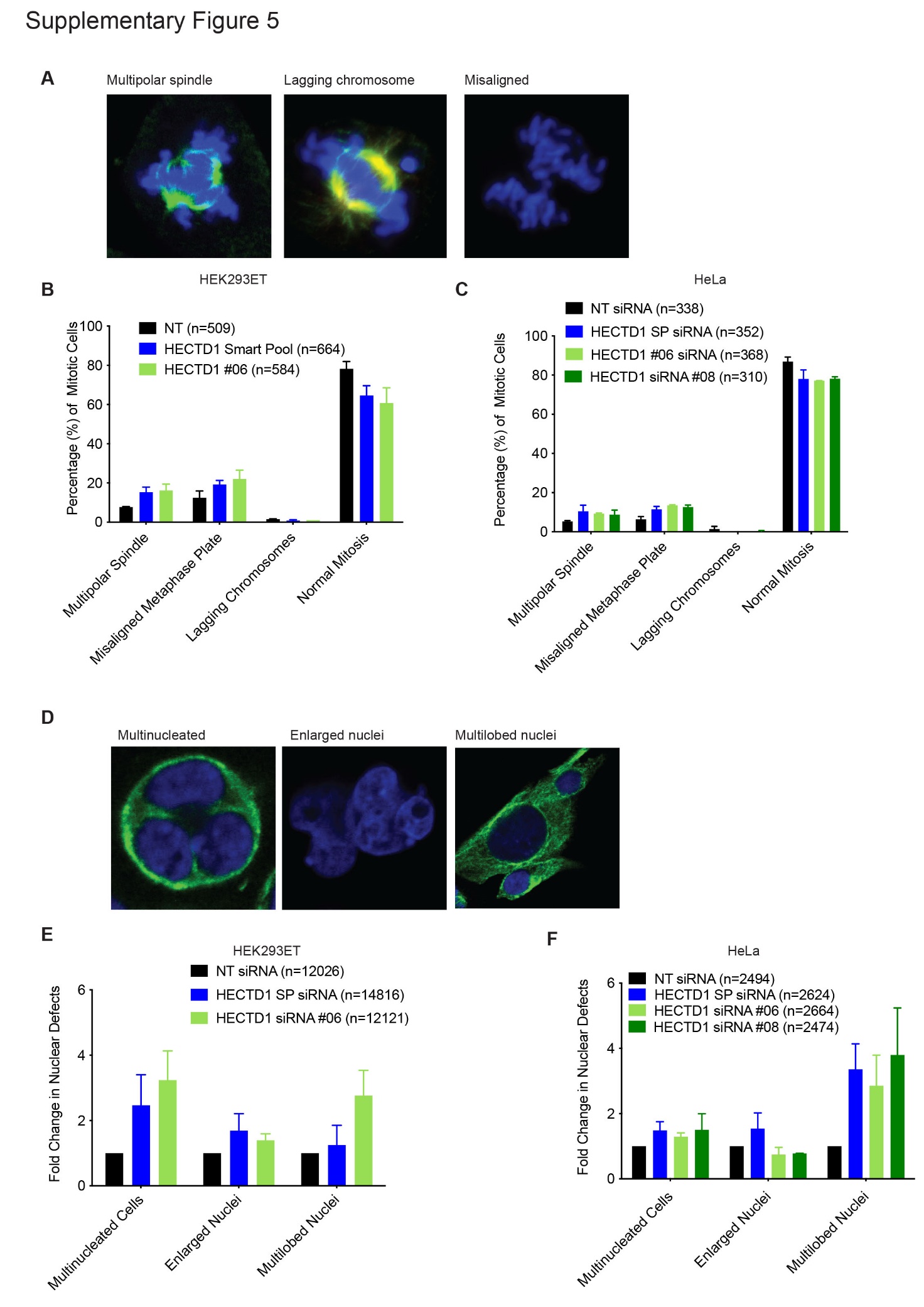


**Supplementary Figure 5. HECTD1-depleted cells show no significant mitotic defect**

**A)** Representative confocal images of mitotic defects scored in HeLa cells. **B**) HEK293ET and **C**) HeLa cells were scored according to chromatin morphology (Hoechst, blue) or spindle morphology based on (α-Tubulin, green) staining following 48 hrs incubation with either NT (non-targeting) siRNA, HECTD1 SMARTpool (SP) siRNA or individual HECTD1 siRNA #06, and #08. Cells were categorised into three mitotic defect phenotypes: multipolar spindle, misaligned metaphase plate, or lagging chromosomes. Cells with no observable mitotic defects were scored as cells that were in normal mitosis. Data plotted as mean with error bars that represent ±S.E.M., over 6 biological repeats. Average increase in multipolar spindle = HECTD1 siRNA of 8.0% HEK293ET; 4.1% HeLa. Average misaligned metaphase plate = HECTD1 siRNA of 7.1% HEK293ET; 6.2% HeLa. However, no statistical significance was found using a one-way ANOVA test. **D**) Representative images of each nuclear defect scored (HeLa) according to chromatin morphology Hoechst (blue). α-Tubulin is shown in green. **E**) HEK293ET and **F**) HeLa cells were scored following 48 hrs incubation with either NT (Non-Targeting) siRNA, HECTD1 SMARTpool (SP) siRNA, individual HECTD1 siRNA #06, and #08. Cells were grouped into three nuclear defect phenotypes: multinucleated cells, enlarged nuclei, or multilobed nuclei. Data plotted as mean with error bars that represent ±S.E.M., over 6 biological repeats. HEK293ET HECTD1 #08 siRNA represents one independent experiment. No statistical significance was found using a one-way ANOVA test.


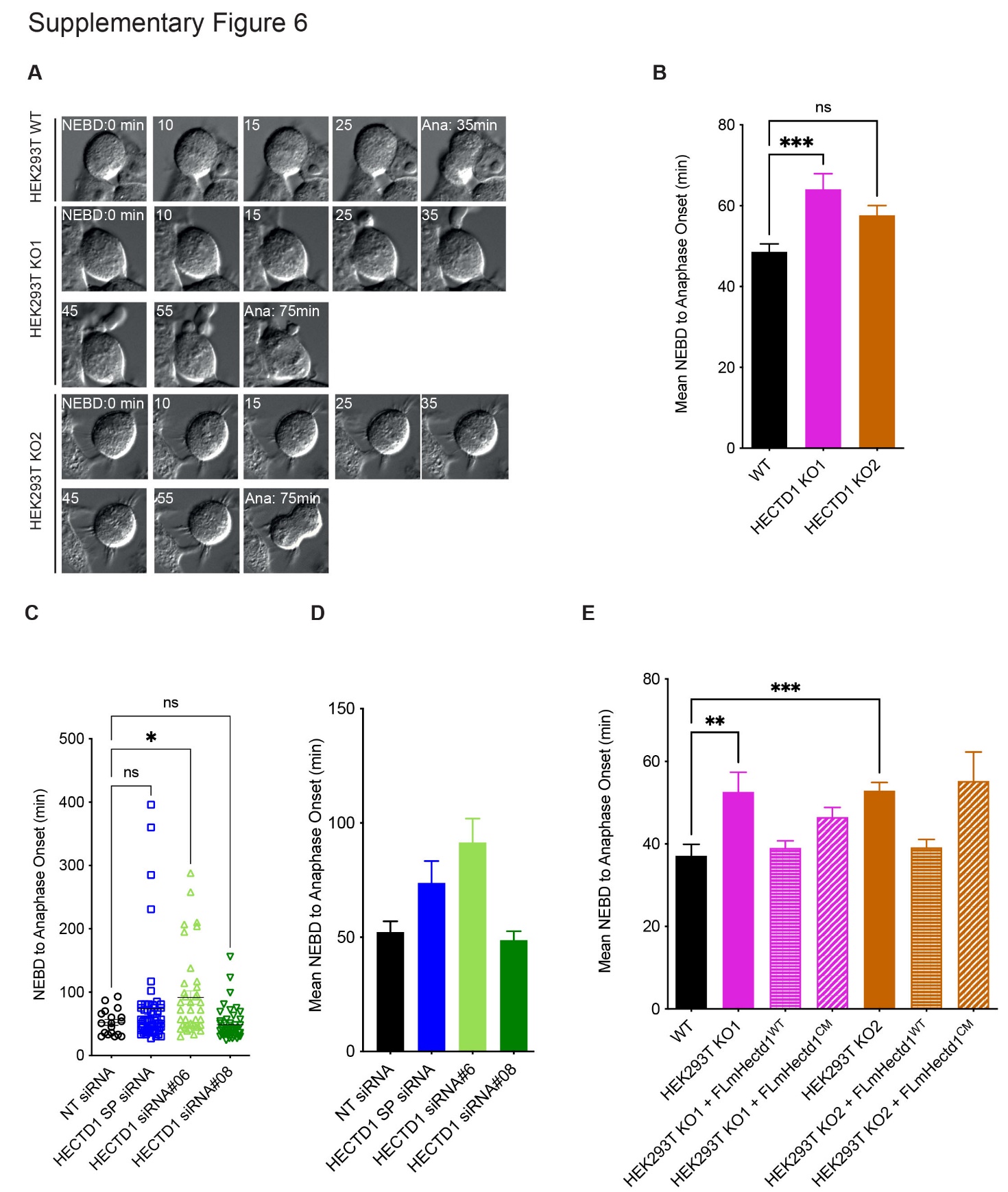


**Supplementary Figure 6. HECTD1 ubiquitin ligase activity contributes to NEBD-to-anaphase onset**

**A**) Representative still images of cells scored in Fig 4D. Cells were imaged using an Olympus IX81 microscope with a 40X oil immersion objective lens and Hammatsu ORCA-ET Camera at 37°C. Micro-Manager was used to acquire and analyse images^3^. Interestingly, the anaphase image of KO1 appeared tripolar, reflecting our earlier data that some HECTD1-depleted cells might have multipolar spindles although this did not reach statistical significance (Supplementary Fig 5B). **B**) Mean time taken (min) for HEK293T wild-type, HECTD1 KO1 and KO2 to progress from NEBD to anaphase onset for data shown in Fig 4D. Error bars represent ±S.E.M., ***p<0.001, using a one-way ANOVA with a Dunnett’s post-test. Number of cells filmed are as follows, WT = 116, KO1 = 136, and KO2 = 161, filmed over 4 independent experiments. **C**) Vertical scatter plot showing the time taken for each cell to progress from NEBD to anaphase onset 72 hrs post transfection of HEK293ET with either Non-Targeting (NT), HECTD1 SP (SMARTpool), individual HECTD1 siRNA#06 or #08. **D**) Mean time taken (min) for cells to progress from NEBD to anaphase onset for data shown in C). Error bars represent ±S.E.M., *p<0.05, by using a one-way ANOVA with a Dunnett’s post-test. Number of cells filmed are as follows, NT (72 hrs) = 18, HECTD1 SP (72 hrs) = 57, HECTD1 siRNA#06 (72 hrs) = 39, and HECTD1 siRNA#08 (72 hrs) = 45, filmed over 3 independent experiments. Similar data were obtained following transfection for 48 hrs (not shown). **E**) Mean time taken (min) for cells to progress from NEBD to anaphase onset for data shown in Fig 4E). Error bars represent ±S.E.M., ***p<0.001, by a one-way ANOVA with a Dunnet’s post-test. Number of cells filmed are as follows, HEK293T WT = 127, KO1 = 97, KO1 + HA-FLmHectd1^WT^ = 48, KO1 + HA-FLmHectd1^CM^ = 108, KO2 = 153, KO2 + HA-FLmHectd1^WT^ = 46, and KO2 + HA-FLmHectd1^CM^ = 19, filmed over 3 independent experiments.


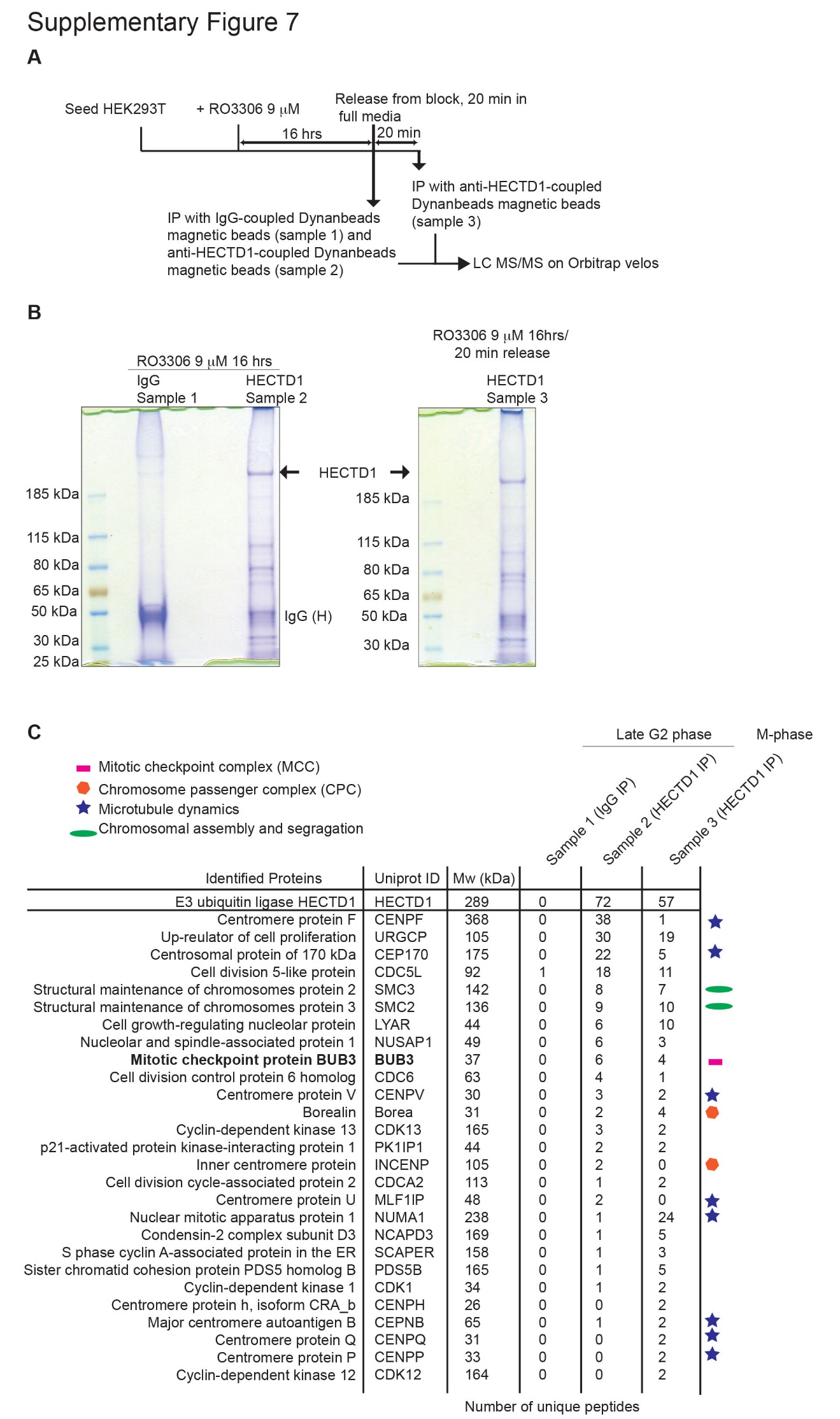


**Supplementary Figure 7. Proof-of-principle study of HECTD1 interactome in late G2 and M-phases**

**A)** Schematic of the proteomics experiments to identify candidate interactors of endogenous HECTD1 in HEK293T. **B**) Coomassie stained gel showing proteins captured using IgG-coupled magnetic beads (G2) or anti-HECTD1-coupled magnetic beads from lysates of cells synchronised in late G2 and M-phase. Each lane was cut in 24 gel slabs which were analysed by LCMS/MS using an Orbitrap velos (n = 1 IP performed for each sample). **C**) Selected curated list of cell cycle related HECTD1 candidate interactors. The full list of proteins is presented in Supplementary Table 1.


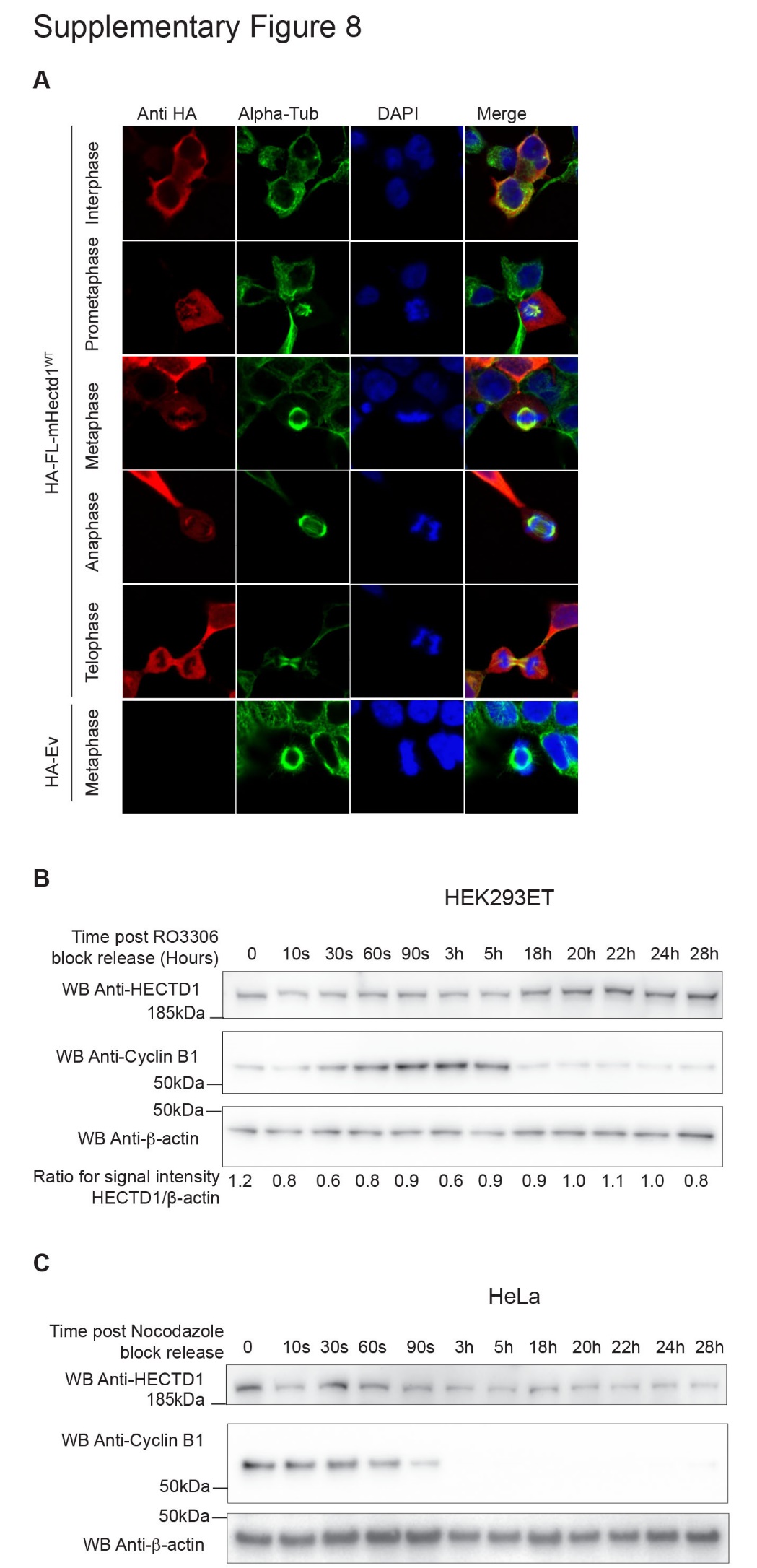


**Supplementary Figure 8. HECTD1 protein levels are not cell cycle regulated.**

**A)** Representative confocal images of HA-FL-mHectd1^WT^ transiently expressed in HEK293ET cells. Cells were transfected with 500 ng of HA-mHectd1^WT^ or HA-tagged empty vector (EV) using PEI, in a 12-well format. 24 hrs post-transfection cells were fixed using 4% PFA, then stained with anti-HA (red), anti-α-tubulin (green), counter stained with Hoechst (blue) and mounted with VectaShield mounting media. Images were taken using an LSM Meta 510 Confocal Microscope. Scale bar represents 10 μm. Images were taken of cells in prometaphase, metaphase, anaphase, and telophase. **B-C)** HECTD1 levels during mitosis were monitored in HEK293ET and HeLa cells synchronised and released from **B)** RO3306 or **C)** nocodazole block, respectively. HEK293ET cells were synchronised with 9 µM RO3306 for 20 hrs prior to release. HeLa cells were synchronised with 50 ng/ml Nocodazole for 20 hrs prior to release. Samples were collected at the indicated time points and analysed by western blot. Cells were lysed in RIPA buffer at the indicated time points post release. Samples were analysed on a 4-12% SDS PAGE and following western blotting on PVDF, the membrane was blocked in 3%-BSA-PBST and incubated overnight with anti-HECTD1 antibody (ab101992) and anti-Cyclin B1 (sc-245), followed by detection with a secondary HRP antibody. Anti-beta-actin (A5441) was used as loading control. (n=1 for each cell synchronisation experiment). Signal intensity was quantified using ImageJ and ratios for pH3(Ser28)/β-actin were determined.


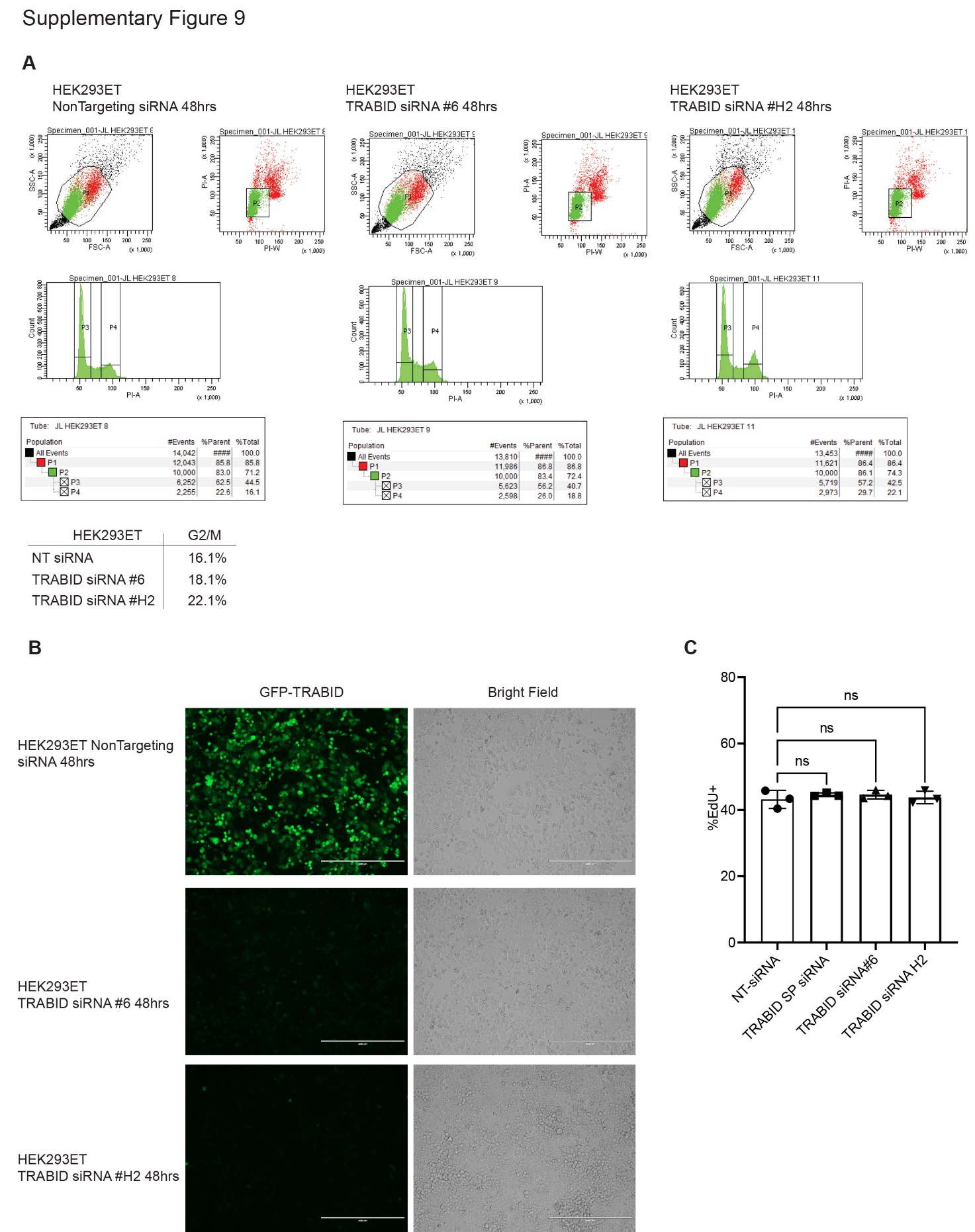


**Supplementary Figure 9. Effect of TRABID transient depletion on cell cycle progression.**

**A)** HEK293ET cells were treated with Non-Targeting (NT) siRNA or TRABID siRNA #6 or #H2 for 48 hrs prior to cell cycle profile analysis by flow cytometry using Propidium Iodide staining (N=1 experiment). **B)** EVOS microscopy images of HEK293ET transfected with a GFP-tagged Full-length TRABID construct together with either NT siRNA, TRABID siRNA #6 or TRABID siRNA #H2 for 48 hrs. Cells were imaged on a EVOS Cell Imaging System (ThermoFisher Scientific). **C)** HEK293T were treated with NT SIRNA, TRABID SMARTpool (SP), TRABID siRNA #6 from SMARTPOOL (SP) or TRABID siRNA #H2. Forty-eight hours following transfections, cells were labelled with Click-EdU staining, images were acquired using an IN CELL Analyser 2000 high-content microscope and analysed as in Figure 2C-F.


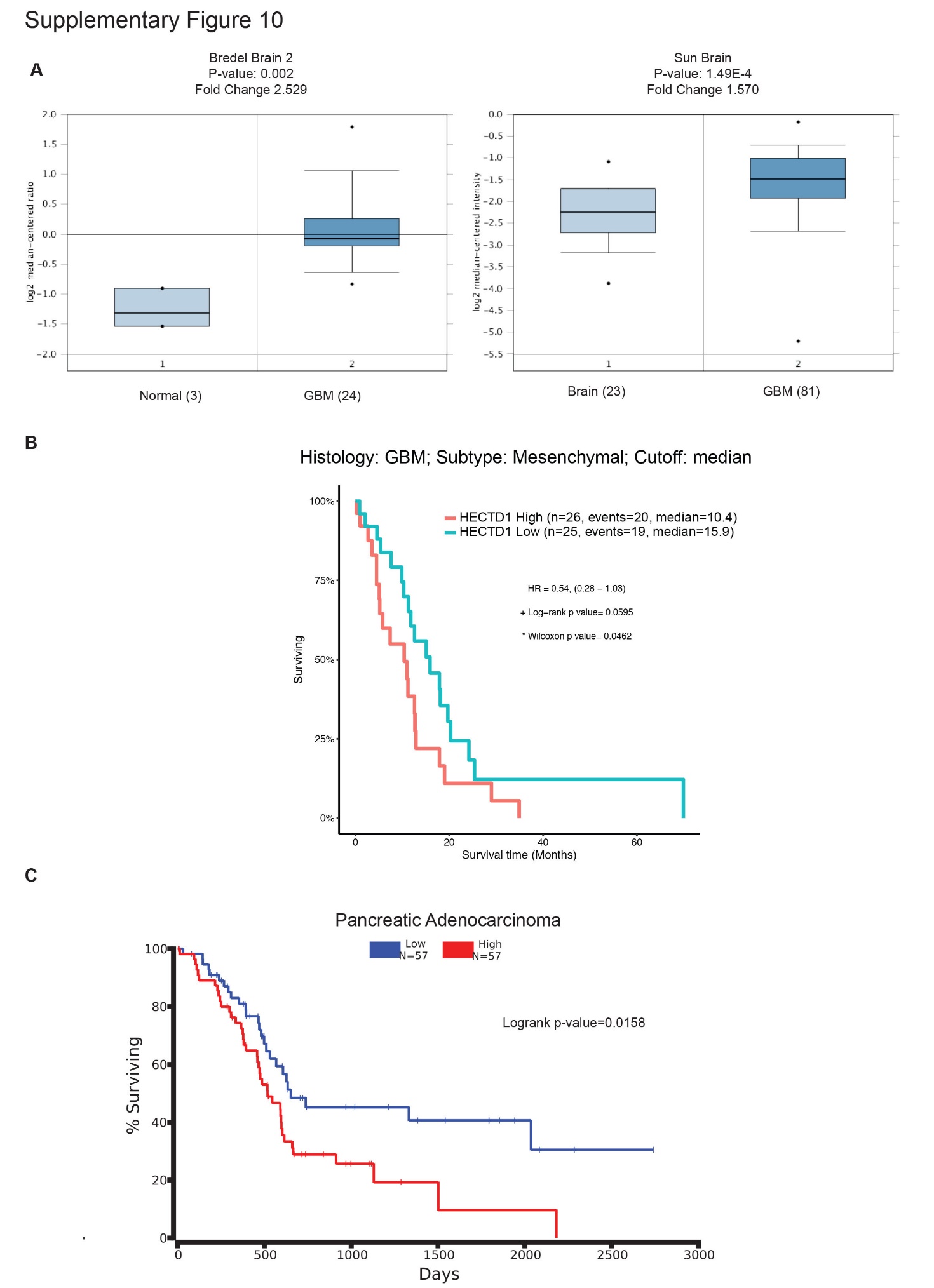


**Supplementary Figure 9. HECTD1 and cancer**

**A)** ONCOMINE (<https://www.oncomine.org/resource/login.html)> showing the fold change in *HECTD1* mRNA levels in normal vs Glioblastoma samples, in the Bredel Brain 2 and the Sun Brain datasets. TCGA analysis suggests high HECTD1 mRNA expression correlates with lower survival time in GBM (Mesenchymal subtype) (**B**) and in pancreatic adenocarcinoma **C**).^4^

**Uncropped western blot membrane images**


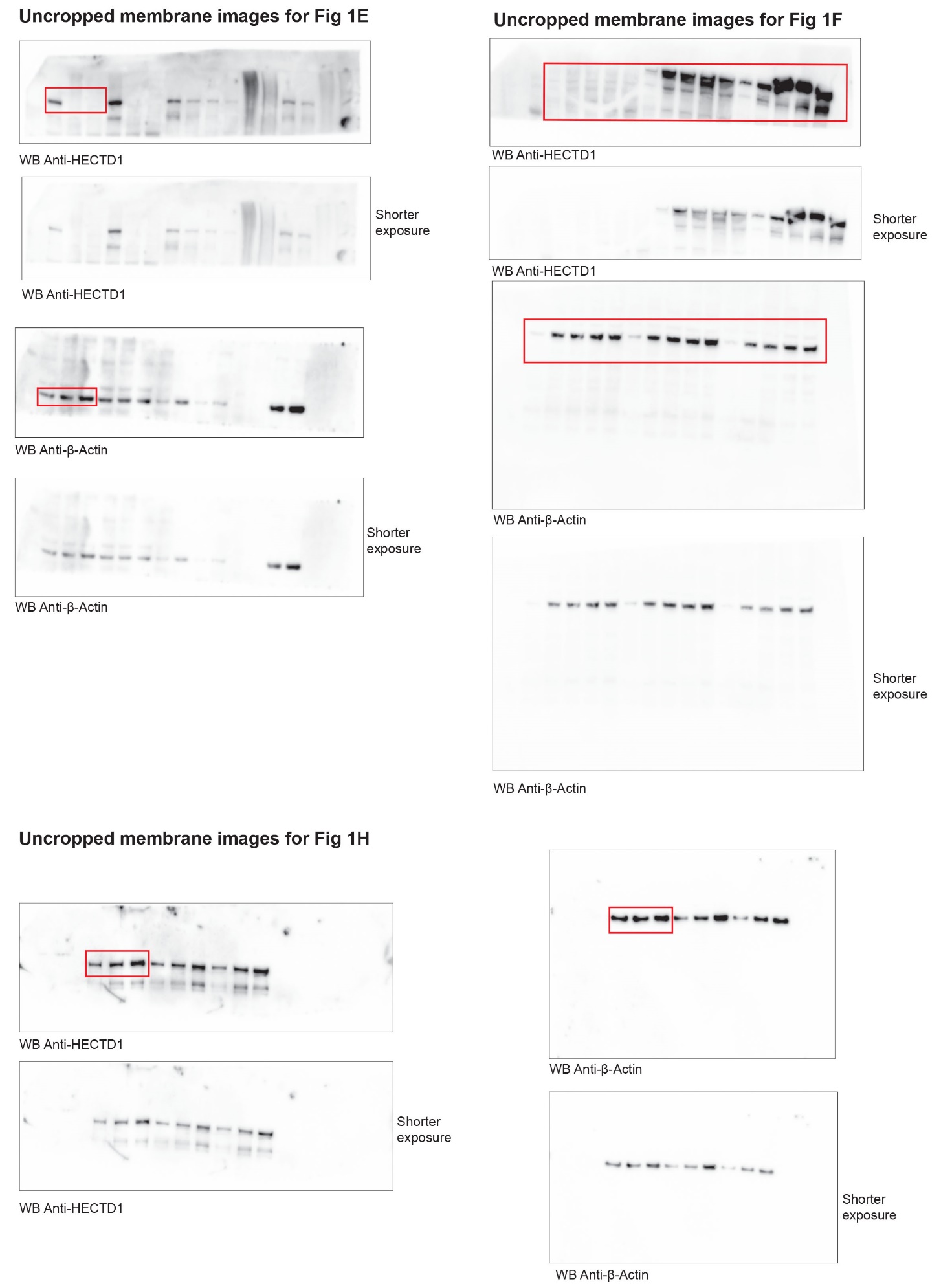


**
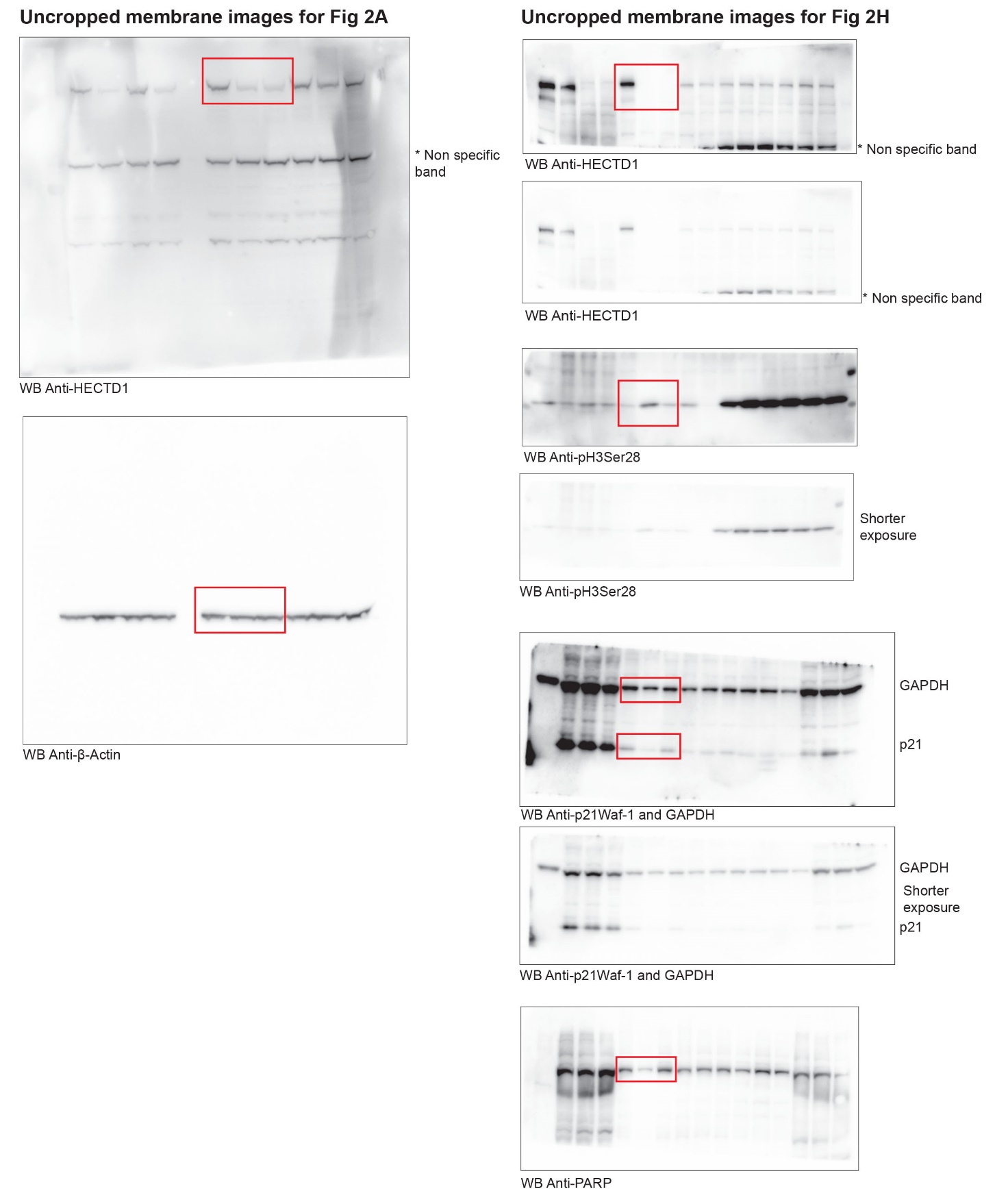
**

**
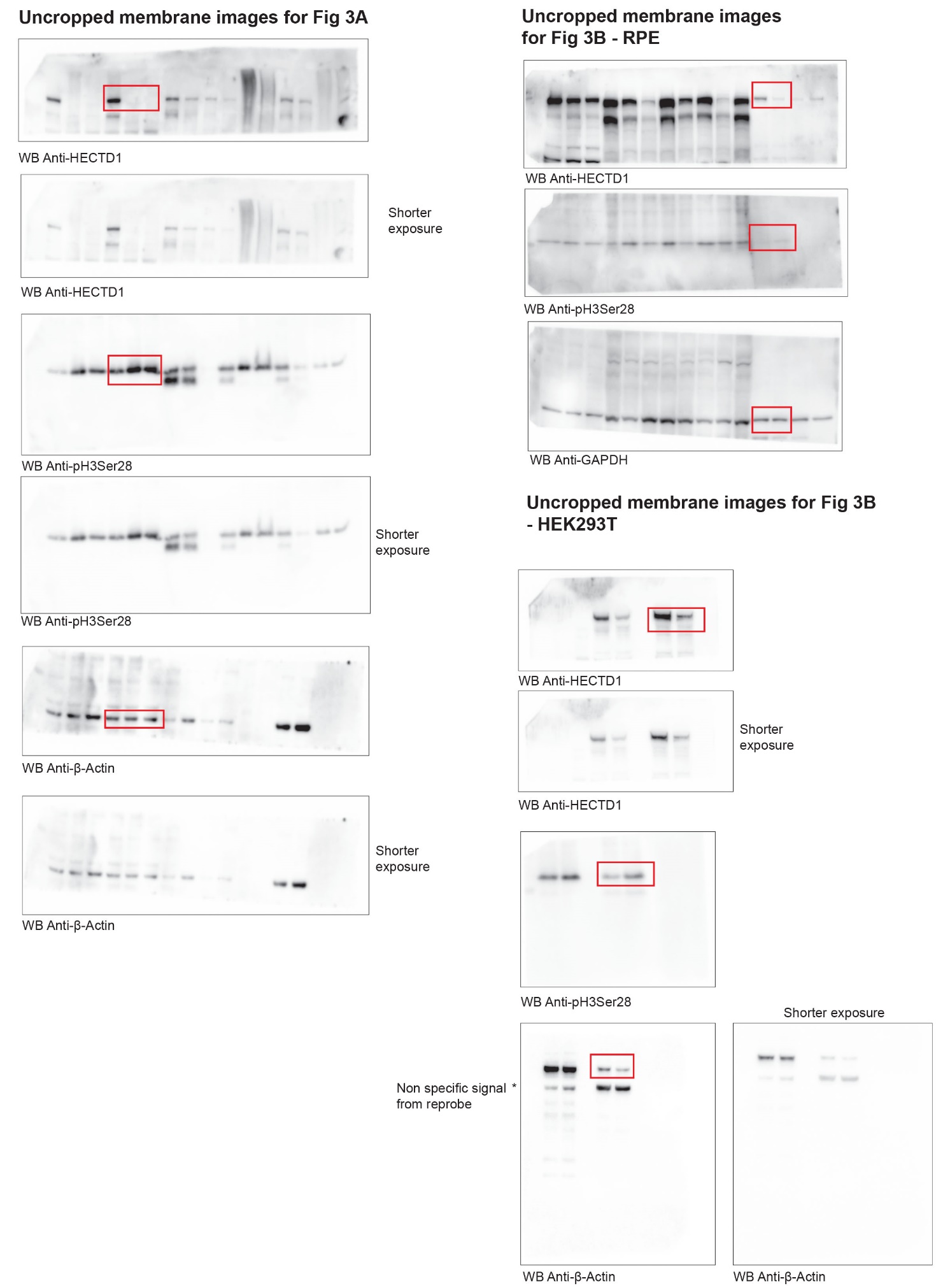
**

**
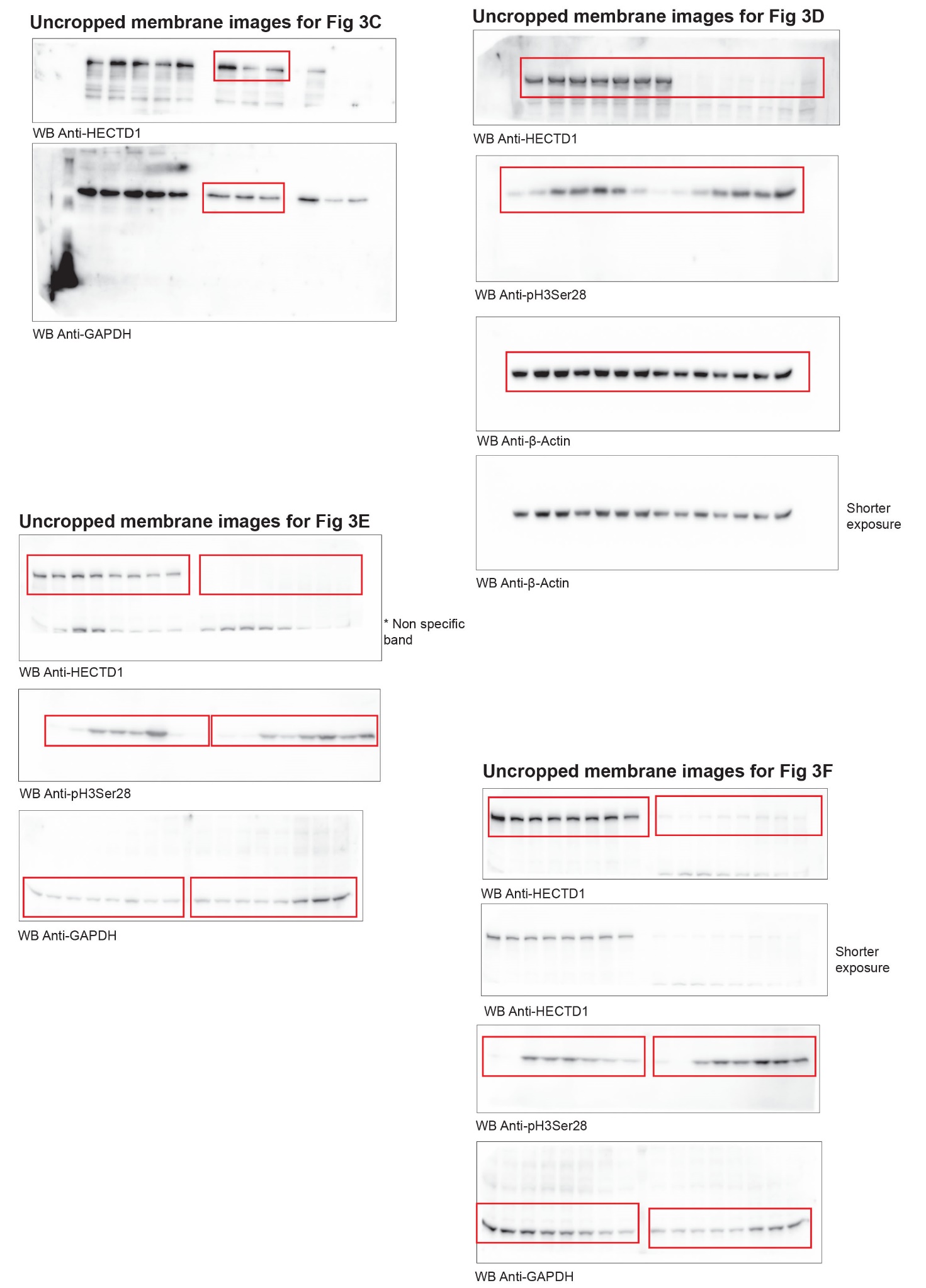
**

**
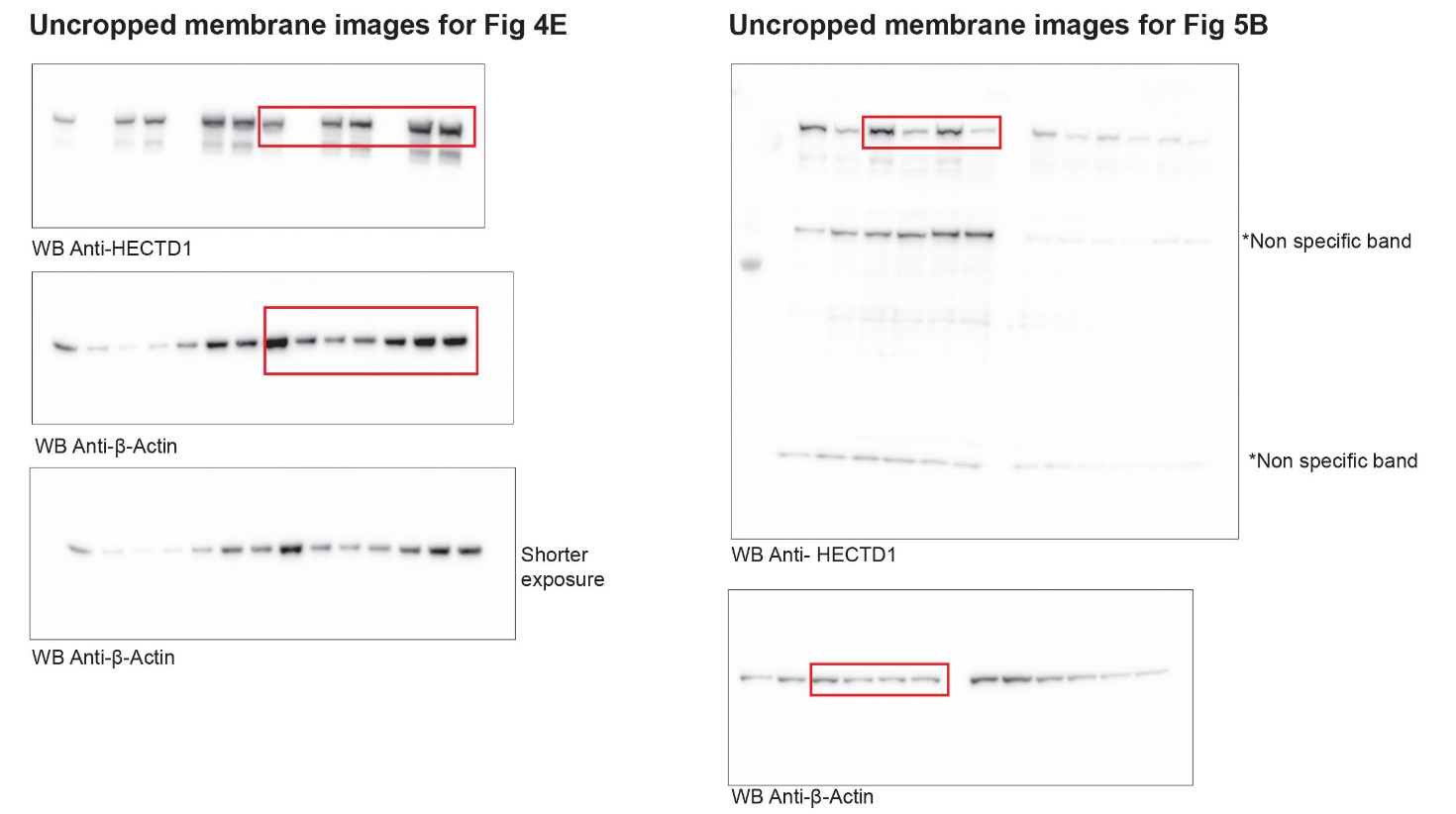
**

**
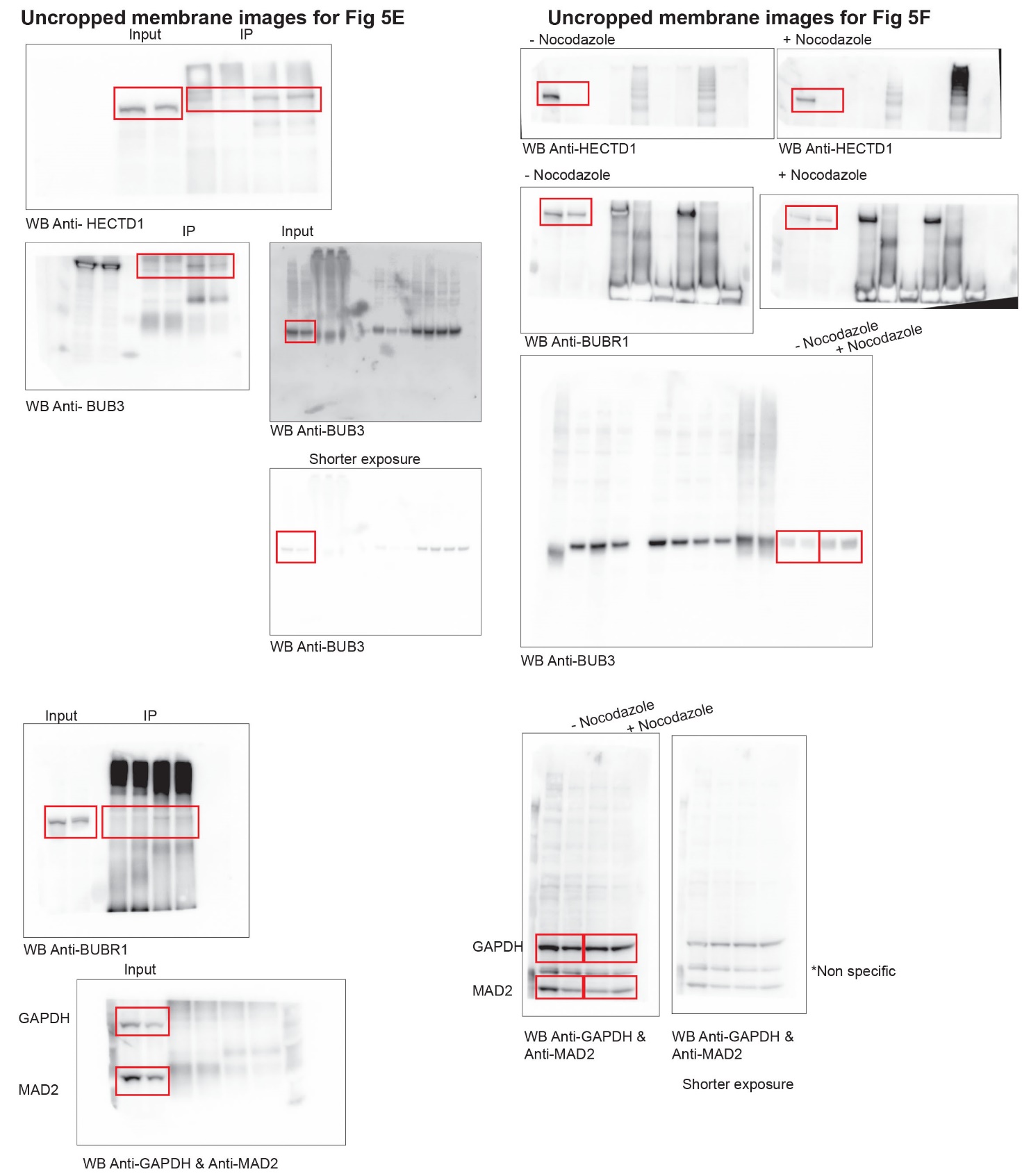
**

**
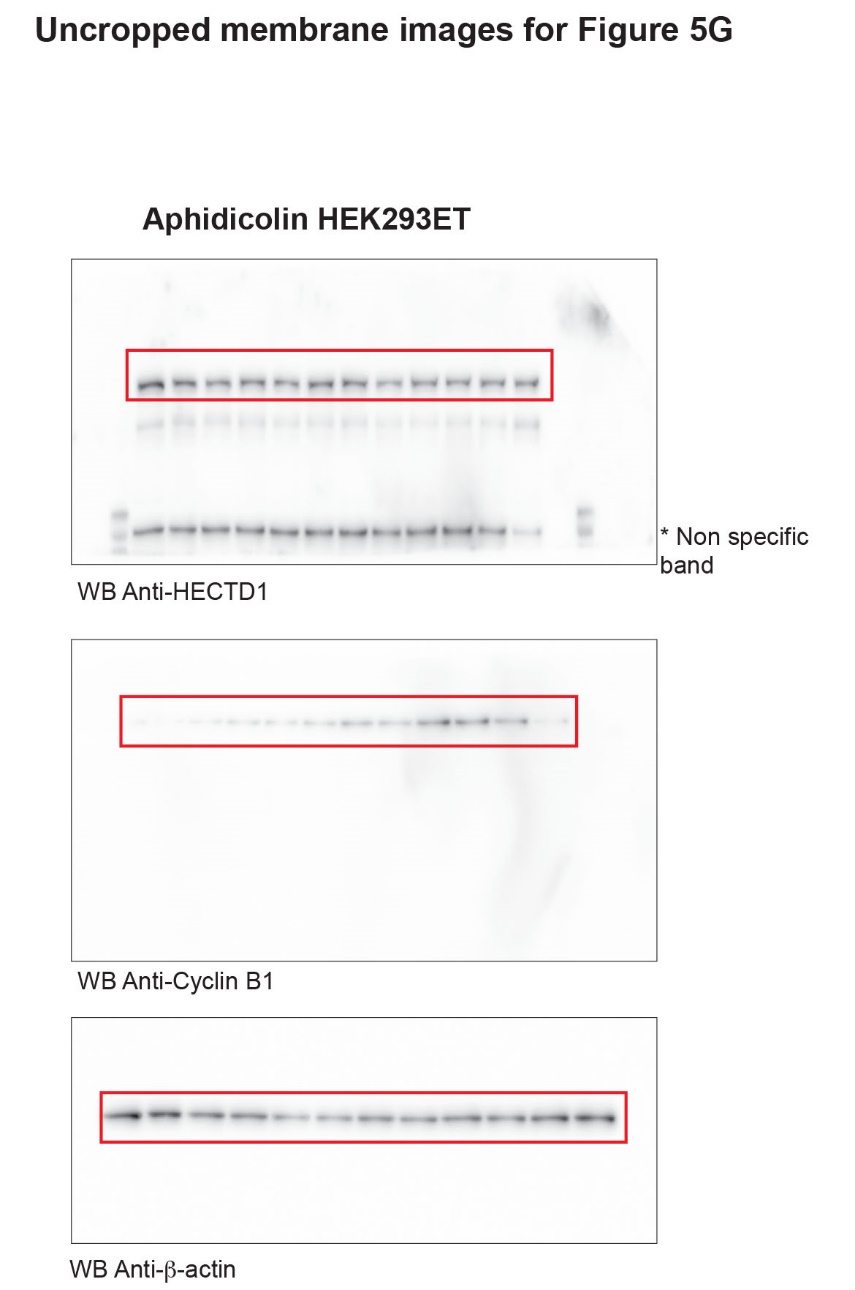
**

**
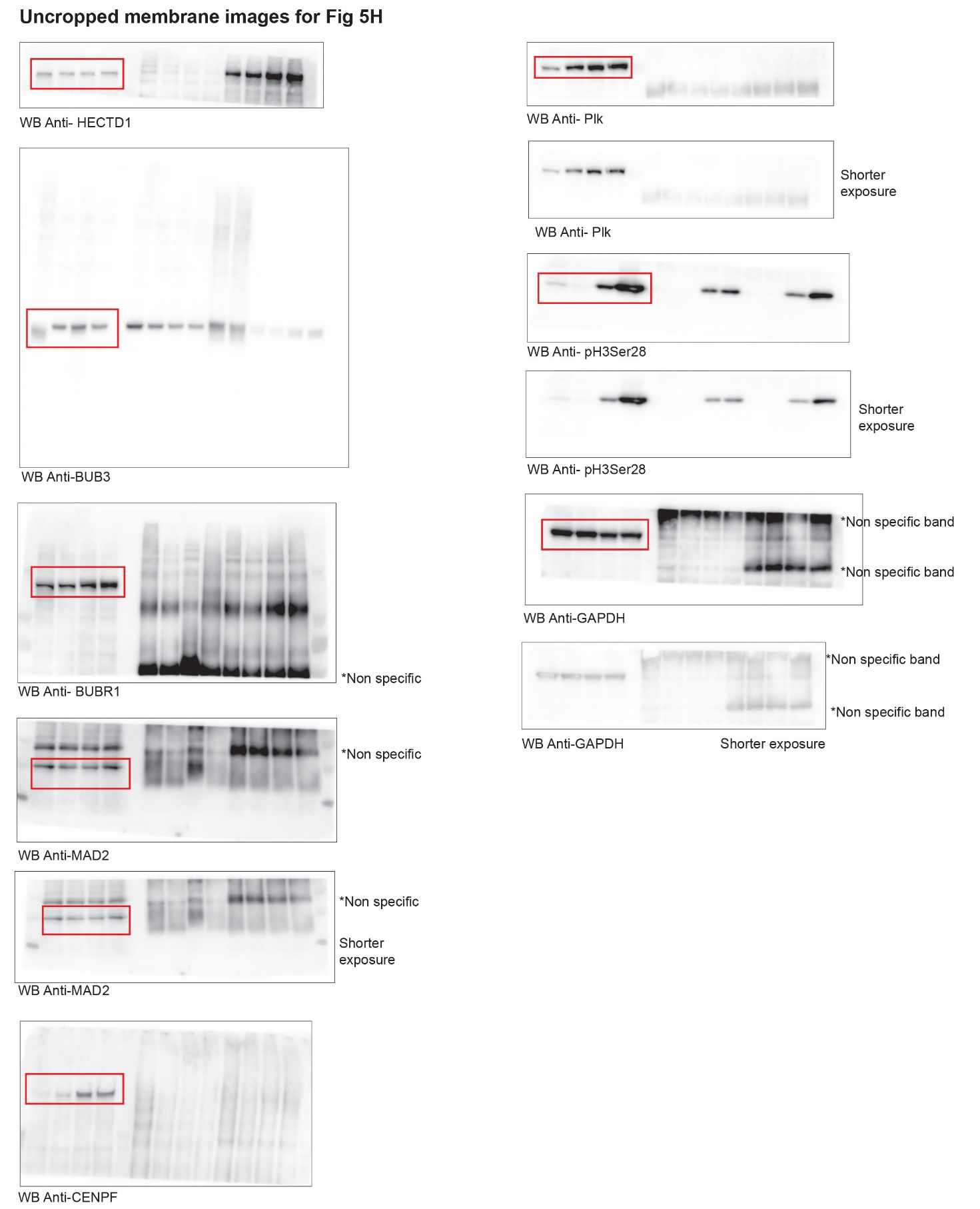
**

**
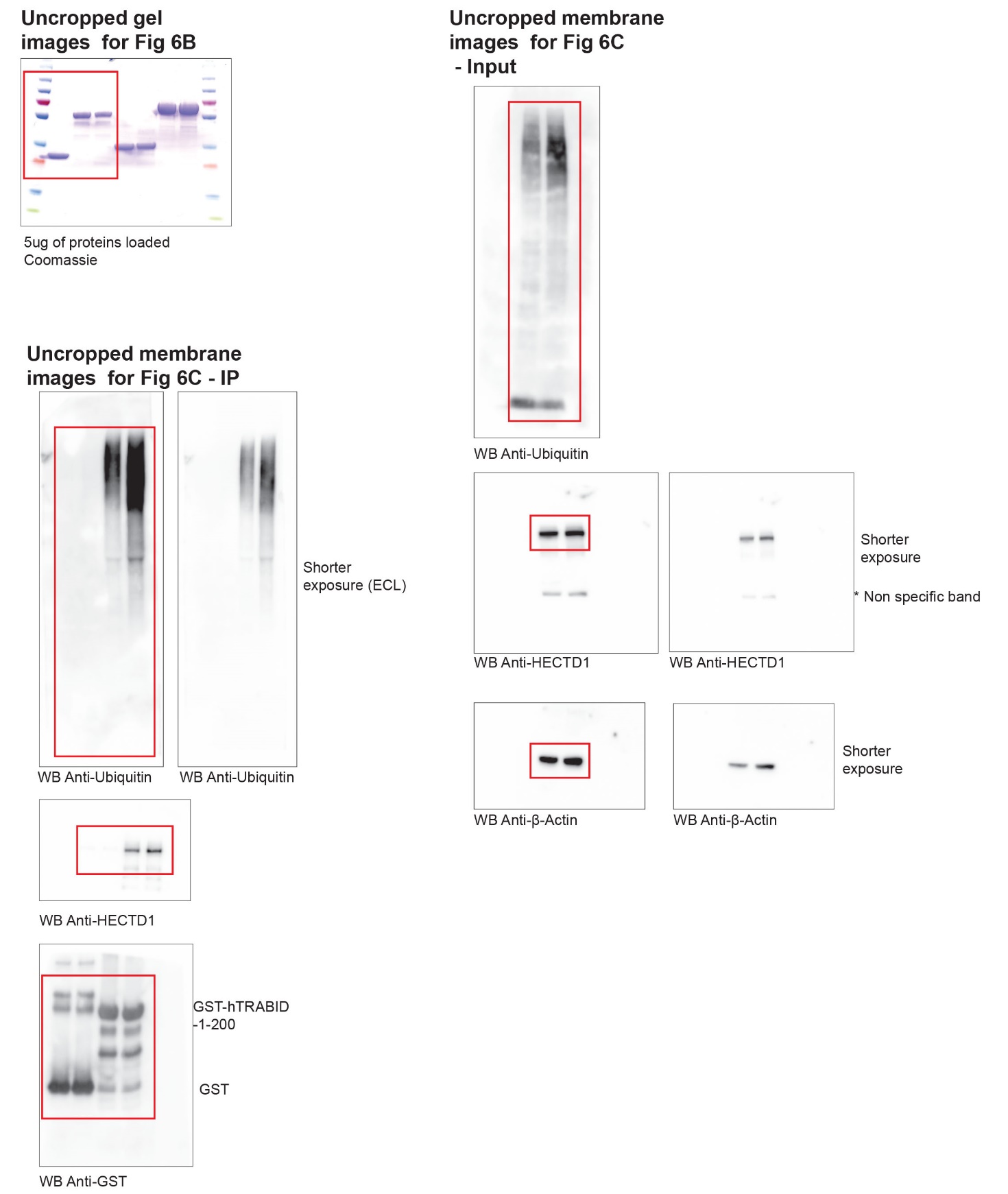
**

**
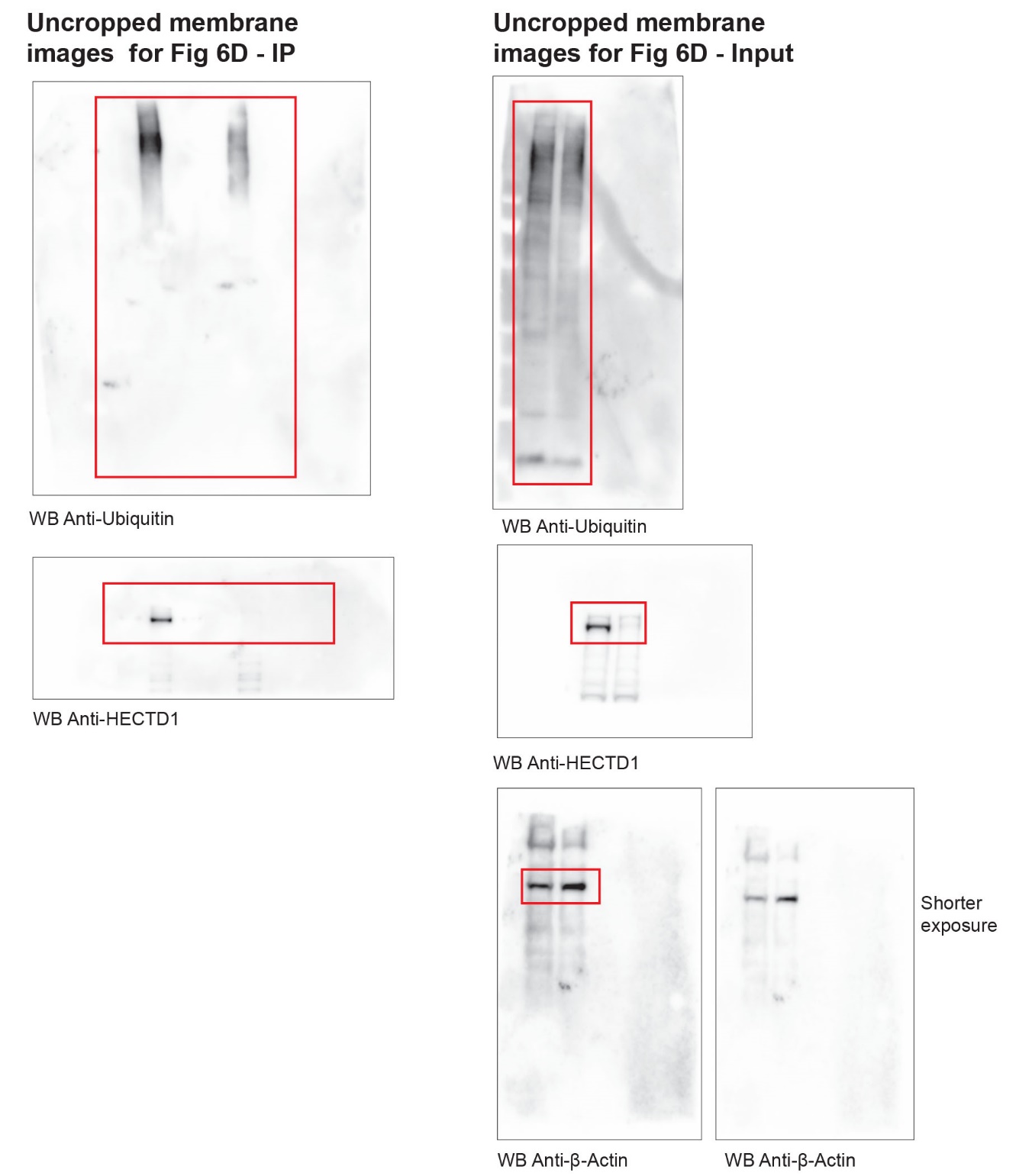
**

**
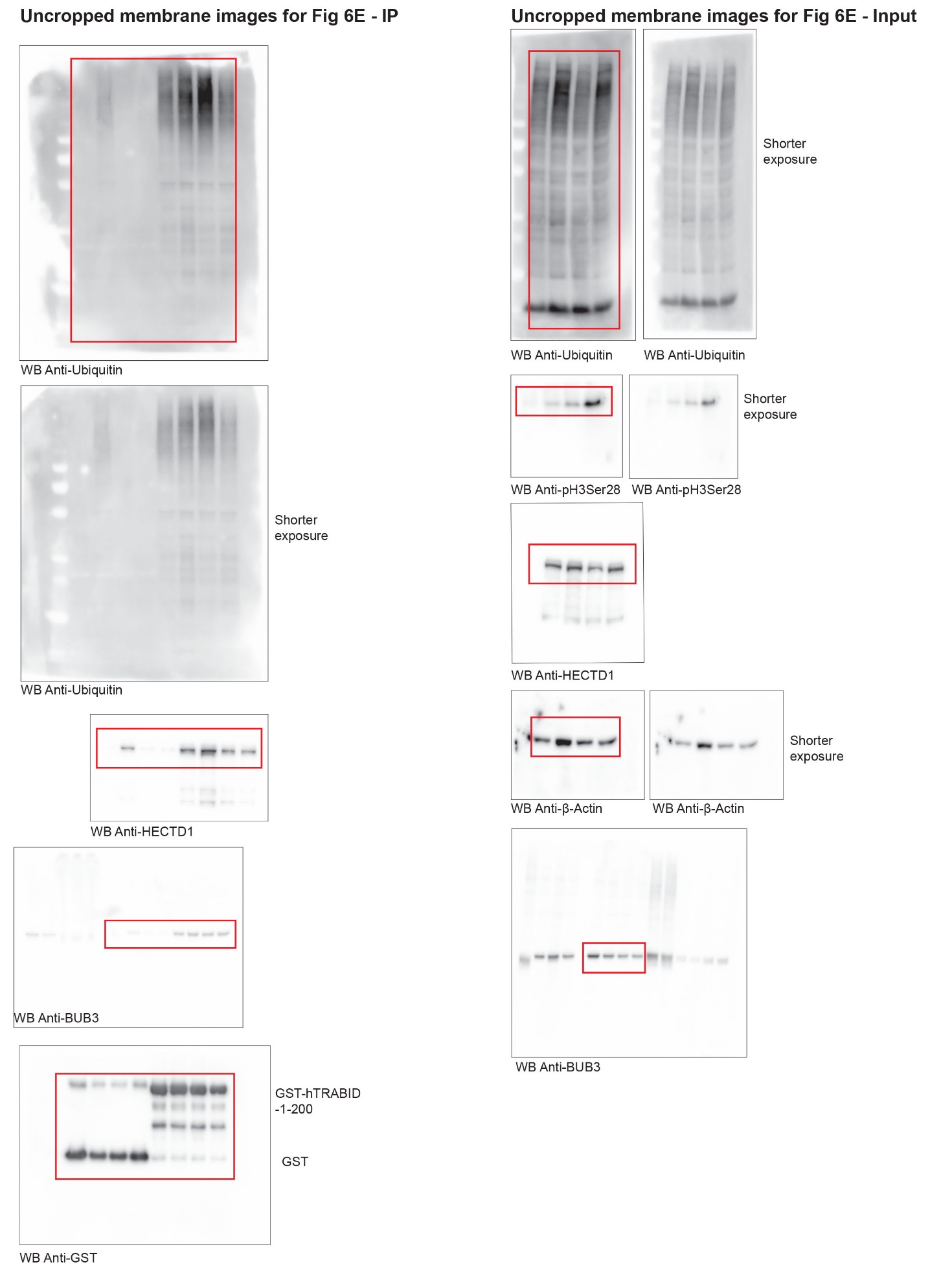
**

**
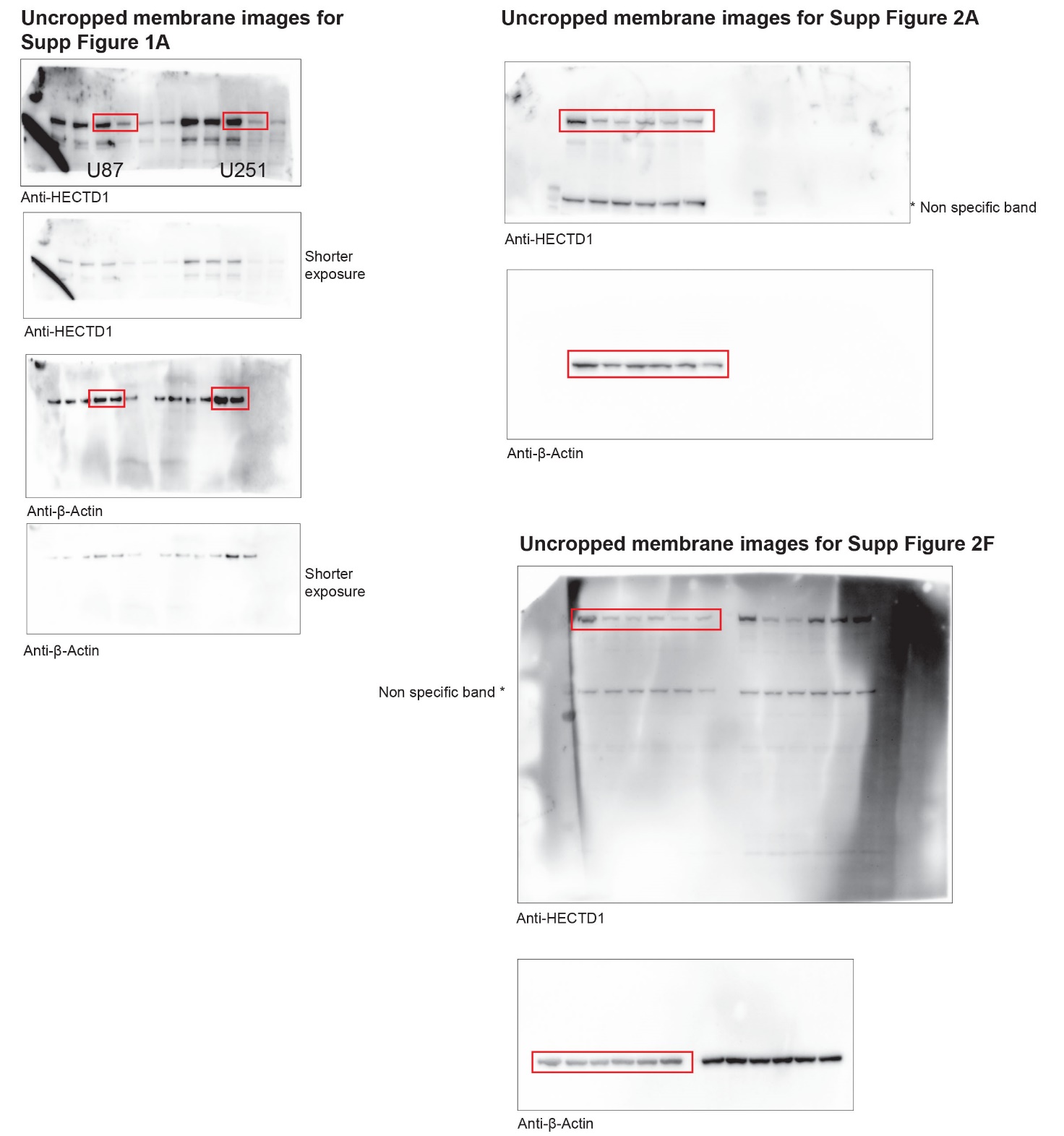
**

**
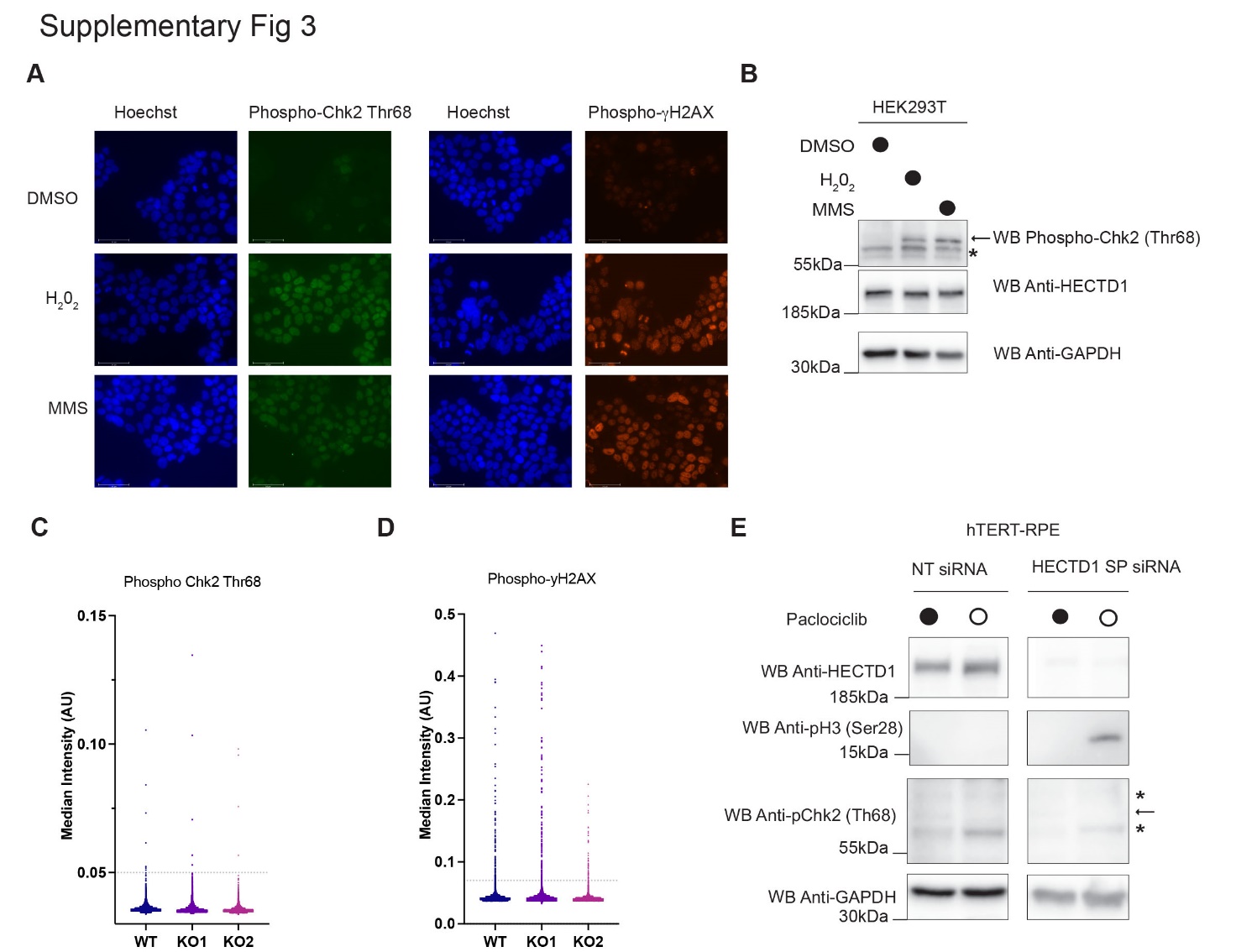
**

**
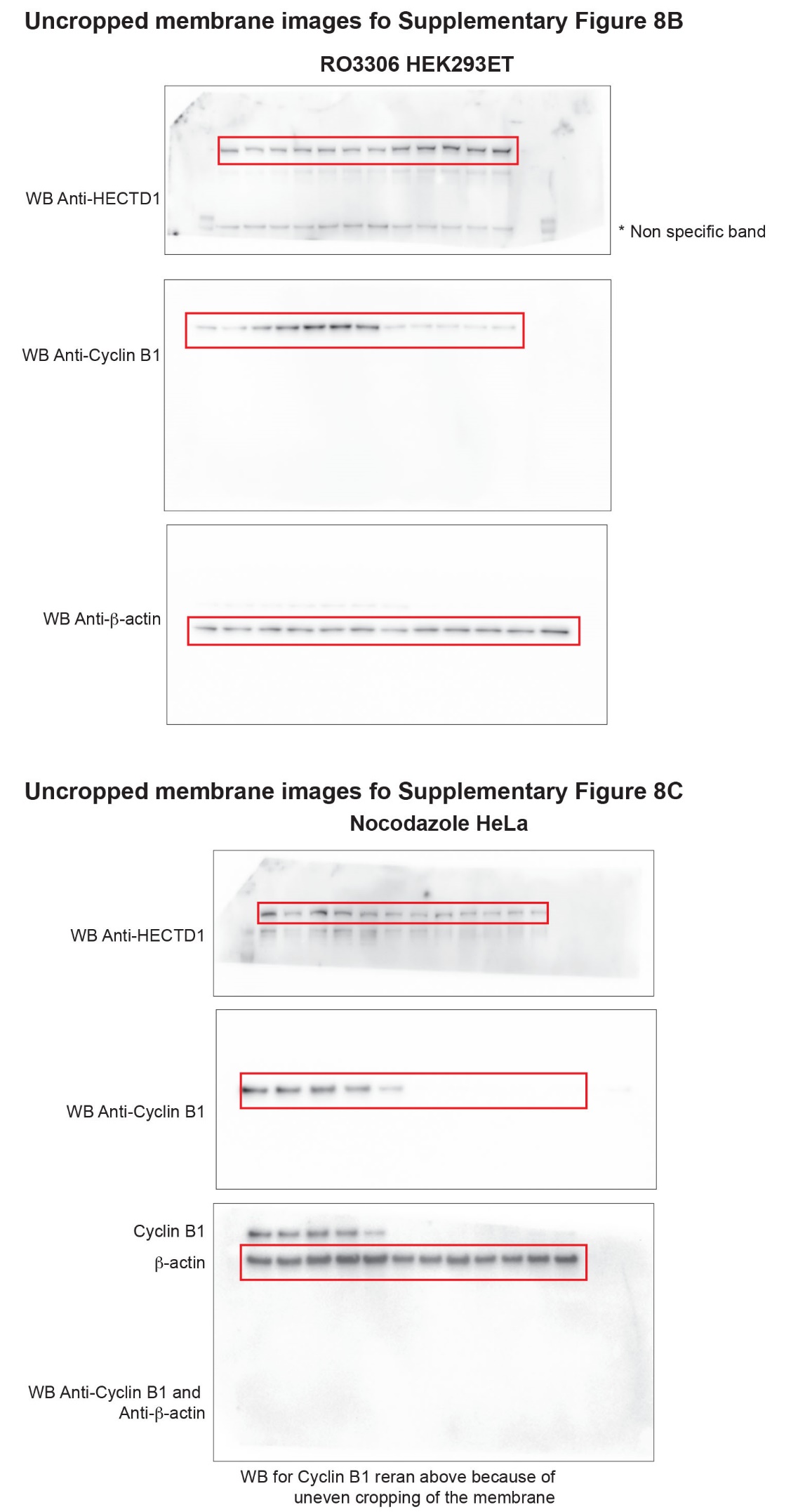
**

**References**

1 Chou, W. C. *et al.* Chk2-dependent phosphorylation of XRCC1 in the DNA damage response promotes base excision repair. *EMBO J* **27**, 3140-3150, doi:10.1038/emboj.2008.229 (2008).

2 Trotter, E. W. & Hagan, I. M. Release from cell cycle arrest with Cdk4/6 inhibitors generates highly synchronized cell cycle progression in human cell culture. *Open Biol* **10**, 200200, doi:10.1098/rsob.200200 (2020).

3 Edelstein, A. D. *et al.* Advanced methods of microscope control using muManager software. *J Biol Methods* **1**, doi:10.14440/jbm.2014.36 (2014).

4 Anaya, J., Reon, B., Chen, W. M., Bekiranov, S. & Dutta, A. A pan-cancer analysis of prognostic genes. *PeerJ* **3**, e1499, doi:10.7717/peerj.1499 (2015).
